# Positive Effects of Education on Cognitive Functioning Depend on Clinical Status and Neuropathological Severity

**DOI:** 10.3389/fnhum.2021.723728

**Published:** 2021-09-09

**Authors:** Michelle G. Jansen, Linda Geerligs, Jurgen A. H. R. Claassen, Eduard J. Overdorp, Inti A. Brazil, Roy P. C. Kessels, Joukje M. Oosterman

**Affiliations:** ^1^Donders Institute for Brain, Cognition and Behavior, Radboud University Nijmegen, Nijmegen, Netherlands; ^2^Department of Geriatric Medicine, Radboudumc Alzheimer Center, Radboud University Medical Center, Nijmegen, Netherlands; ^3^Department of Psychiatry, Gelre Medical Centre, Zutphen, Netherlands; ^4^Department of Medical Psychology, Radboudumc Alzheimer Center, Radboud University Medical Center, Nijmegen, Netherlands; ^5^Vincent van Gogh Institute for Psychiatry, Venray, Netherlands

**Keywords:** education, Alzheimer’s dementia, mild cognitive impairment, subjective cognitive decline, cognitive functioning

## Abstract

**Background:** Variability in cognitive functions in healthy and pathological aging is often explained by educational attainment. However, it remains unclear to which extent different disease states alter protective effects of education. We aimed to investigate whether protective effects of education on cognition depend on (1) clinical diagnosis severity, and (2) the neuropathological burden within a diagnosis in a memory clinic setting.

**Methods:** In this cross-sectional study, we included 108 patients with subjective cognitive decline [SCD, median age 71, IQR (66–78), 43% men], 190 with mild cognitive impairment [MCI, median age 78, IQR (73–82), 44% men], and 245 with Alzheimer’s disease dementia (AD) [median age 80, IQR (76–84), 35% men]. We combined visual ratings of hippocampal atrophy, global atrophy, and white matter hyperintensities on MRI into a single neuropathology score. To investigate whether the contribution of education to cognitive performance differed across SCD, MCI, and AD, we employed several multiple linear regression models, stratified by diagnosis and adjusted for age, sex, and neurodegeneration. We re-ran each model with an additional interaction term to investigate whether these effects were influenced by neuropathological burden for each diagnostic group separately. False discovery rate (FDR) corrections for multiple comparisons were applied.

**Results:** We observed significant positive associations between education and performance for global cognition and executive functions (all adjusted *p-*values < 0.05). As diagnosis became more severe, however, the strength of these associations decreased (all adjusted *p-*values < 0.05). Education related to episodic memory only at relatively lower levels of neuropathology in SCD (β = −0.23, uncorrected *p* = 0.02), whereas education related to episodic memory in those with higher levels of neuropathology in MCI (β = 0.15, uncorrected *p* = 0.04). However, these interaction effects did not survive FDR-corrections.

**Conclusions:** Altogether, our results demonstrated that positive effects of education on cognitive functioning reduce with diagnosis severity, but the role of neuropathological burden within a particular diagnosis was small and warrants further investigation. Future studies may further unravel the extent to which different dimensions of an individual’s disease severity contribute to the waxing and waning of protective effects in cognitive aging.

## Introduction

The process of aging is accompanied by alterations in cognitive functions, ranging in severity from normal aging-related changes, to subjective cognitive decline (SCD), mild cognitive impairment (MCI) and, ultimately, dementia (Jack et al., 2018; Salthouse, 2019). Within each diagnostic group, however, there is considerable inter-individual variability in the extent to which cognitive decline manifests itself (Cabeza et al., 2018; Soldan et al., 2020). Educational attainment is a major contributor to this heterogeneity, where individuals with higher levels of education are not only at a lower risk of developing cognitive impairments (Livingston et al., 2020), but also demonstrate better cognitive performance than those with lower educational attainment, sometimes even in advanced stages of pathological aging (Lövdén et al., 2020; Seblova et al., 2020; Stern et al., 2020). The extent to which the positive effects of education are sustained across SCD, MCI, and Alzheimer’s disease dementia (AD) remains poorly understood. It has been suggested that disease state may attenuate such effects (Stern, 2012; Gregory et al., 2017; Mungas et al., 2021), and previous studies have demonstrated that the benefits of higher education become less pronounced or disappear entirely as disease severity increases (Ye et al., 2013; Soldan et al., 2015; Groot et al., 2018). In contrast, recent findings revealed stronger education-cognition associations in AD relatively to SCD and MCI (Staekenborg et al., 2020). Furthermore, neuropathological burden varies greatly within each diagnostic group (Mehta and Schneider, 2021), and the protective effects of education may consequently vary as a function of neuropathological severity (Perneczky et al., 2009; Mungas et al., 2018, 2021).

In this cross-sectional study, we therefore investigated how different syndromal states that vary in neuropathological severity may alter the positive effects of education on cognitive functions in a memory clinic population. More specifically, we investigated whether this relationship differed (1) *across* diagnostic categories with varying levels of clinical severity (SCD, MCI, and AD), and (2) *within* each diagnostic category based on the severity of neuropathological features.

## Materials and Methods

### Study Population

For this retrospective study, we included a total of 543 participants; 108 with SCD, 190 with MCI, and 245 with dementia due to AD. Data from this study sample were extracted from a database containing data of patients who were referred for memory complaints to the memory clinic at Gelre Hospital in Zutphen, Netherlands, between November 2004 and February 2015. All participants underwent a comprehensive clinical and neurological evaluation, neuropsychological assessment, blood screening, electroencephalogram, and magnetic resonance imaging (MRI; Overdorp et al., 2014). Patients were excluded from the present study if MRI data were missing and/or of too poor quality for assessment.

Each clinical diagnosis was established within multidisciplinary consensus meetings and in accordance with the established criteria. Diagnosis of MCI was based on the Petersen et al. (2001) criteria. Diagnosis of probable AD was based on the DSM-IV-TR criteria for dementia of the Alzheimer’s type (APA, 2000). Although assessment of cerebrospinal fluid (CSF) biomarkers is not part of the standard diagnostic work-up in the Netherlands for diagnosing AD, CSF biomarkers were additionally obtained as supportive evidence in case no consensus was reached. Those patients who neither demonstrated any cognitive impairments after neuropsychological assessment using age- and education adjusted normative data, nor suffered from a psychiatric or neurological disorder, were classified as having SCD.

### Education

Educational attainment was measured using the Dutch education classification system, which distinguishes different educational *levels*, rather than using the *years* of education that are typically used in the Anglo-Saxon world. This educational classification is comparable with the International Standard Classification of Education (UNESCO, 2011), and results in a score between 1 and 7: (1) unfinished primary school, (2) finished primary school, (3) unfinished low-level secondary education, (4) lower vocational training, (5) advanced vocational training or lower professional education, (6) finished higher professional education or senior general secondary education, and (7) obtained a university degree (Verhage, 1964). Considering the low prevalence of individuals with unfinished primary school (*n* = 7) and a university degree (*n* = 31), we made the categorical distinction between low (Verhage scores 1–3), average (4–5), and high education level (6–7; Zhou et al., 2019).

### Cognitive Functioning

All neuropsychological tests were administered and subsequently analyzed by two experienced neuropsychologists, that were blinded to all medical records at the time. A detailed overview of the assessment protocol and tests has been described previously (Overdorp et al., 2014). For the present study, we only included tests with available normative data. Briefly, we incorporated the total score of the Mini-Mental State Examination (MMSE; Folstein et al., 1975), the total score of the Visual Association Test (VAT; Lindeboom et al., 2002), the immediate and delayed recall of the 8-Word Test of the Amsterdam Dementia Screening (de Jonghe et al., 1994), the total number correct of the Semantic/Verbal Fluency Test (1-min animal/profession naming), the total score of the Frontal Assessment Battery (FAB; Dubois et al., 2000), the time to complete part A of the Trail Making Test (TMT; Reitan, 1958), and the TMT ratio score (time to complete B/A).

We generated compound scores for three cognitive domains: global cognition, episodic memory, and executive functioning. First, raw test scores were z-standardized using the mean and standard deviation of the whole study sample. We inverted the z-scores of the TMT, so that higher z-scores are always indicative of better cognitive performance. Compound scores were then calculated for each cognitive domain by taking the average of the z-scores from the available (sub-)tasks of an individual corresponding to that domain. Global cognition was based on scores of the MMSE, VAT, 8-Word Test, Verbal Fluency, FAB, TMT A, and TMT ratio; episodic memory on the VAT and 8-Word test; executive functioning on Verbal Fluency, FAB, and TMT ratio. If participants were unable to complete part B of the TMT (*N* = 112), which primarily occurred in patients with MCI (*N* = 40) and AD (*N* = 65), the lowest possible z-score of the sample was assigned. Participant characteristics for the individual (sub-)test scores, including an overview of missing data, are displayed in Supplementary Table A.

### MRI

All MRI scans were obtained using a 1.5 Tesla GE-Signa Horizon LX scanner. Briefly, the MRI protocol included the following sequences: whole brain axial and coronal fluid-attenuated inversion recovery (FLAIR) sequences (TR/TE 10.000/160 ms); a sagittal T1-weighted sequence (TR/TE 300/4 ms); and an axial T2-weighted sequence (TR/TE 6,500/105 ms).

### Measures of Neuropathology

Three experienced independent observers (EO, JC, and JO), blinded to the clinical diagnoses and neuropsychological test scores, visually rated white matter hyperintensities (WMH), medial temporal lobe atrophy (MTA), and global atrophy (GA). In this study, we employed qualitative visual rating scales as these are easily applicable in clinical practice and, relatively to volumetric measures, provide comparable or even more reliable assessments of neuropathology (Gouw et al., 2006; Persson et al., 2018; Topiwala et al., 2019). WMH were rated on axial FLAIR and T2-weighted images using the Fazekas scale, providing a score between 0–3 based on the deep and periventricular areas of the brain (Fazekas et al., 1987). MTA was rated on coronal T1-weighted images with a 5-point (0–4) scale, considering the height of the hippocampus as well as the width of the choroid fissure and temporal horn of the left and right MTA (Scheltens et al., 1992). GA was rated on a 4-point (0–3) rating scale using all available MRI sequences, and represented the mean score for cortical atrophy based on the width of gyri and sulci across the whole cerebrum (Scheltens et al., 1997).

As we were interested in capturing the accumulation of neuropathological damage rather than the effects of a particular type of neuropathology, we combined the effects of MTA, GA, and WMH to obtain a single measure indicative of neuropathology per patient in relation to each separate cognitive domain. To accomplish this, first, separate multiple linear regression models were performed using the cognitive domain scores as dependent variables and the measures of neuropathology as predictors. As sample sizes differed across the diagnostic groups, with substantially less cases of SCD, we wanted to make sure that our neuropathology metric was not biased by the number of patients per group. To this end, a bootstrap scheme was adopted. Across 100 replications, we randomly selected 75 cases from each diagnosis group. Within each bootstrap, leave one out cross validation (LOOCV) was applied to retrieve optimal model parameters. The resulting intercepts and regression weights were averaged to obtain the final parameters, and were subsequently inverted to retrieve the final measure of neuropathological burden (the higher this score, the more neurodegeneration was present that was relevant to a particular cognitive domain). These final burden scores allowed us to identify whether cognitive-domain specific neuropathology scores affect the relationship between education and cognitive functioning.

### Statistical Analysis

Demographics, vascular risk factors, cognitive performance and measures of brain degeneration were compared between groups using univariate tests (analysis of variance, ANOVA; Chi-squared test; Mann–Whitney *U* test; and Kruskall–Wallis test, where appropriate). *Post hoc* pairwise comparisons (SCD vs. MCI; SCD vs. AD; MCI vs. AD) were corrected for multiple comparisons using false discovery rate (FDR) adjustments.

First, to investigate whether the contribution of education to cognitive performance differed *across* the different clinical diagnoses (SCD, MCI, and AD), we applied Analysis of Covariance (ANCOVA) models. The cognitive domain scores functioned as outcome variables and education level, diagnostic group, and the interaction between education level and diagnostic group as predictors. Subsequently, to further investigate the direction of significant interactions, several multiple linear regression models were performed stratified by diagnostic group, and the resulting slopes of education were compared pairwise using Welch *T*-tests (SCD vs. MCI, SCD vs. AD). All analyses were corrected for age, sex, and neuropathology scores.

Second, we investigated whether the effects of education differed as a function of neuropathology *within* each diagnosis group. We performed another set of multiple linear regression models, but now separately for each diagnosis group, and education level, neuropathology, and the interaction between education and neuropathology functioned as predictors. This model allowed to test whether the relationship between education and cognitive performance was moderated by current degree of neuropathology. Significant interactions were further examined using simple slope analysis from the *interactions* package in R (Bauer and Curran, 2005). Briefly, the relationship between education and cognitive performance was plotted as a function of different degrees of relative neuropathological burden: lower levels of neuropathology [−1 standard deviation (SD)], average neuropathology (0 SD), and higher levels of neuropathology (+ 1 SD).

Furthermore, we performed sensitivity analyses to investigate whether our results were influenced by using standardized norm scores to calculate the cognitive domain scores, as norm scores provide an indication of cognitive performance relatively to an individual’s age, sex and/or educational level. Norm scores were computed using a large Dutch normative database from the Advanced Neuropsychological Diagnostics Infrastructure (ANDI; de Vent et al., 2016). As normative data for the FAB were unavailable in ANDI, these norm scores were generated using another normative database (Coen et al., 2016). Moreover, given that floor performance on the TMT ratio scores and delayed recall of the 8-Word Test occurred more frequently in AD and MCI relatively to SCD (see Supplementary Table A), we also repeated our analyses while deriving the compound scores without these particular (sub-)tests. In addition, we investigated whether the effects of the neuropathological compound score were driven by a particular MRI rating (MTA, GA, or WMH). Therefore, we repeated our analysis using either MTA, GA, or WMH in the interaction term and corrected for the other neuropathological features (e.g., interaction between education and MTA, additionally correcting for GA and WMH).

For each linear regression model, all variables were scaled (i.e., z-normalized) prior to the analysis. Assumptions were checked using regression diagnostic plots and the *gvlma* package in R (Pena and Slate, 2006). None of the assumptions were violated (e.g., linearity, distribution of residuals, homoscedasticity).

All analyses were performed using R (version 3.6.1^1^). Two-tailed *p*-values of < 0.05 were considered statistically significant. We report uncorrected *p*-values and FDR-corrected *p*-values to account for multiple comparisons across diagnoses and cognitive domains. We calculated Cohen’s f^2^ to indicate the effect sizes for our effects of interest (0.02 = small, 0.15 = medium, 0.35 = large; Cohen, 2013). Data visualization was performed using the *raincloudplots* and *sjPlot* packages in R (Allen et al., 2021; Lüdecke, 2021).

## Results

**FIGURE 1:**
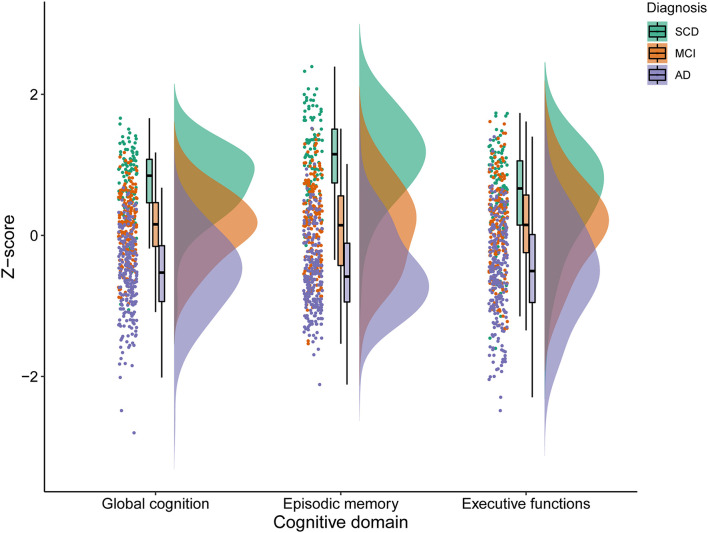
Variability in cognitive performance in SCD, MCI, and AD. SCD, subjective cognitive decline; MCI, mild cognitive impairment; AD, Alzheimer’s disease dementia.

An overview of participant characteristics for participants with SCD, MCI, and AD is provided in Table 1. The variability in cognitive performance across each cognitive domain and diagnosis group is visualized in Figure 1. We did not observe any differences between diagnostic groups regarding sex (*p* = 0.11), diabetes (*p* = 0.80), hypertension (*p* = 0.56), cardiac disease (*p* = 0.06), and history of stroke (*p* = 0.91). After FDR-adjustments for multiple comparisons, age, education, cognitive performance and visual MRI ratings differed significantly between groups (all corrected *p-*values < 0.01). *Post hoc* tests revealed that individuals with SCD were relatively younger than those with MCI or AD, and those with MCI were younger than with AD (all corrected *p*-values < 0.05). Educational attainment and cognitive performance scores were relatively higher in SCD, followed by MCI and AD (all corrected *p*-values < 0.05). MTA, GA, and WMH were less pronounced in individuals with SCD when compared to both MCI and AD; and both MTA and GA were less severe in MCI relatively to AD (all corrected *p*-values < 0.05).

**TABLE 1:** Overview of participant characteristics.

	SCD	MCI	AD	
*N*	108	190	245	*p*-value
**Demographics**				
Age, median (IQR)	71 (66–78)	78 (73–82)	80 (76–84)	< 0.001^a,b,c,d^
Sex, *N* (%)	61 (57%)	106 (56%)	159 (65%)	0.110
Education level, median (IQR)	5 (3–5)	4 (4–5)	4 (3–5)	0.006^a,c,d^
High education, *N* (%)	26 (24%)	46 (24%)	45 (18%)	
Average education, *N* (%)	54 (50%)	102 (54%)	114 (47%)	
Low education, *N* (%)	28 (26%)	42 (22%)	86 (36%)	
**Vascular risk factors**				
Diabetes mellitus, *N* (%)	20 (19%)	30 (16%)	39 (16%)	0.800
Hypertension, *N* (%)	46 (43%)	88 (46%)	101 (41%)	0.561
History of stroke/TIA, *N* (%)	16 (15%)	30 (16%)	35 (14%)	0.915
Cardiac disease, *N* (%)	26 (24%)	67 (35%)	66 (27%)	0.063
**Cognitive functions**				
MMSE, mean (SD)	28.06 (2.02)	26.25 (2.49)	22.34 (3.84)	< 0.001^a,b,c,d^
Global cognition, mean (SD)	0.74 (0.49)	0.13 (0.47)	−0.56 (0.58)	< 0.001^a,b,c,d^
Episodic memory, mean (SD)	1.13 (0.58)	0.10 (0.63)	−0.52 (0.58)	< 0.001^a,b,c,d^
Executive functions, mean (SD)	0.56 (0.69)	0.12 (0.63)	−0.51 (0.72)	< 0.001^a,b,c,d^
**Neuropathological measures**				
MTA, median (IQR)	0 (0–1)	1 (1–2)	2 (1–2)	< 0.001^a,b,c,d^
WMH, median (IQR)	1 (0−2)	2 (1–3)	2 (1–3)	< 0.001^a,b,c^
GA, median (IQR)	1 (0–1)	1 (1–2)	1 (1–2)	< 0.001^a,b,c,d^

*SCD, subjective cognitive decline; MCI, mild cognitive impairment; AD, Alzheimer’s disease dementia; TIA, transient ischemic attack; MTA, medial temporal lobe atrophy; WMH, white matter hyperintensities; GA, global atrophy. Information was missing for history of stroke/TIA in 1 (0.18%), cardiac disease in 1 (0.18%), episodic memory in 2 (0.37%), executive functions in 1 (0.18%). *P*-values displayed are uncorrected.*

*^a^Group contrast, surviving FDR-correction for multiple comparisons.*

*^b^Significant SCD vs. MCI comparison after FDR-corrections.*

*^c^Significant SCD vs. AD comparison after FDR-corrections.*

*^d^Significant MCI vs. AD comparison after FDR-corrections.*

**FIGURE 2:**
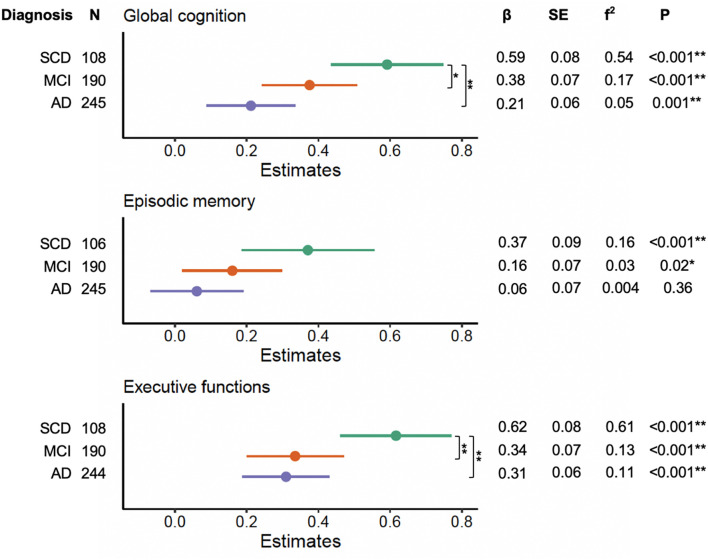
Effects of education on cognitive performance across diagnosis severity. Forest plots indicating the role of education in predicting cognitive performance across diagnosis groups, corrected for age, sex, and neuropathological burden. Effect sizes for the contribution of education were calculated with Cohen’s f^2^. *P*-values displayed are uncorrected. Differences in slopes (β) between diagnosis groups were compared using Welch’s *t*-tests (SCD vs. MCI, SCD vs. AD, MCI vs. AD). SCD, subjective cognitive decline; MCI, mild cognitive impairment; AD, Alzheimer’s disease dementia. *Uncorrected *p* < 0.05. **FDR-corrected *p* < 0.05.

We noted significant interactions between education and clinical diagnosis on global cognition (*F_2,534_* = 4.48, *p* = 0.01), episodic memory (*F_2,532_* = 3.84, *p* = 0.01), and executive functions (*F_2,533_* = 5.09, *p* = 0.006). Forest plots of the subsequent stratified multiple linear regression models, by diagnostic group, corrected for age, sex, and neuropathological burden, are displayed in Figure 2. These analyses revealed that education independently contributed to cognitive performance on global cognition and executive functions across each diagnosis group (all corrected *p-*values < 0.05), but not on episodic memory in MCI and AD (all corrected *p-*values > 0.05). Statistical comparison of the corresponding slopes showed that the associations between education and global cognition were stronger in those with SCD relatively to both MCI and AD (uncorrected *p* = 0.04 and *p* = 0.002, respectively). However, the slope difference between SCD and MCI did not survive FDR-corrections (corrected *p* > 0.05). For executive functions, we found that the effects of education were stronger in the SCD group as compared to both MCI and AD (all corrected *p*-values < 0.05).

**FIGURE 3:**
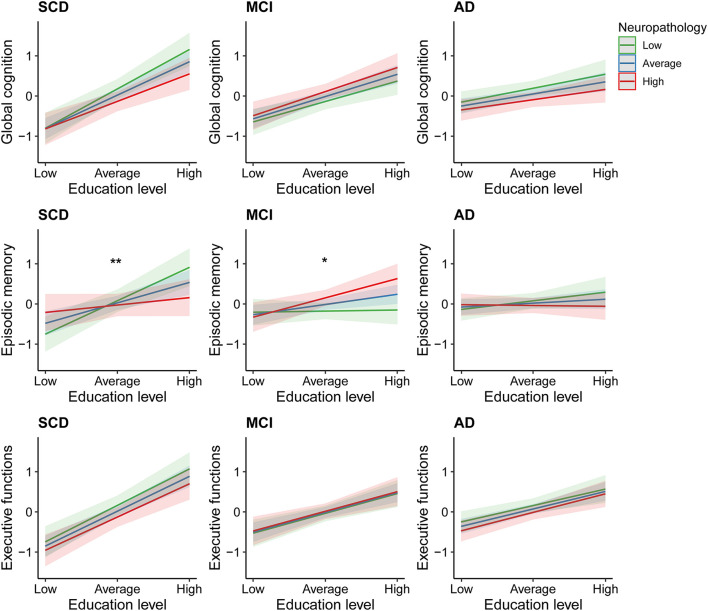
Effects of education on cognitive performance across different degrees of neuropathological burden. SCD, subjective cognitive decline; MCI, mild cognitive impairment; AD, Alzheimer’s disease dementia. *Uncorrected *p* = 0.04 for interaction between education and neuropathological burden. **Uncorrected *p* = 0.02 for interaction between education and neuropathological burden.

The second set of linear regression models, modeling the interaction between education and neuropathological burden, revealed significant interactions in the domain of episodic memory only. These were found among those individuals with SCD [β = −0.23, 95% CI = (−0.42; −0.04), uncorrected *p* = 0.02], and MCI [β = 0.15, 95% CI = (0.01; 0.30), uncorrected *p* = 0.04; Figure 3]. However, these associations did not survive FDR-corrections. In the SCD group, subsequent simple slope analyses revealed significant effects of education on episodic memory only in those with average or relatively lower levels of neuropathology (β = 0.36, uncorrected *p* < 0.001; β = 0.59, uncorrected *p* < 0.001). In contrast, among patients with MCI, simple slope analyses revealed that education only significantly contributed to episodic memory performance in those with average or relatively higher levels of neuropathology (β = 0.17, uncorrected *p* = 0.02; β = 0.33, uncorrected *p* = 0.003). Results from the multiple linear regression models, including and excluding the interaction terms, are provided in Supplementary Table B.

The sensitivity analyses showed that the use of standardized norm scores rather than whole population z-scores to calculate the cognitive domain scores concurred with weaker effects of education on cognitive performance across the different diagnoses. However, the direction of results remained similar (i.e., effect of education on cognition decreased with diagnosis severity). Moreover, the interaction between education and neuropathology on episodic memory in MCI was no longer significant (see Supplementary Table C and Supplementary Figure A). After calculating the compound scores without the TMT ratio and 8-Word Test delayed recall, our results remained largely similar, however the interaction between education and neuropathology on episodic memory was no longer significant in MCI (see Supplementary Table D). When using the separate visual MRI ratings in our models, our results were also largely unaffected. We observed that MTA was the main driver of the interaction effect in SCD, whereas GA was the main driver of the interaction in MCI. A complete overview of these results is shown in Supplementary Tables E, F, G.

## Discussion

In this study, we characterized the effects of education on cognitive functions across different syndromes that vary in the severity of neuropathology and cognitive dysfunction. First, we focused on the different clinical diagnoses as observed in a memory clinic population, comparing patients with SCD, MCI and AD. We demonstrated that the contribution of education in explaining variability in cognitive performance decreased with diagnosis severity, independent of age, sex, and neuropathological burden. Second, we concentrated on the severity of neuropathological burden within these diagnostic groups. We found that the role of education differed as a function of neuropathology in the domain of episodic memory. In SCD, the effects of education on cognition were only found in those individuals with lower levels of neuropathology. In contrast, among those with MCI, the role of education was merely present in individuals with relatively more severe neuropathological burden.

The observed positive effects of education on cognitive functioning throughout SCD, MCI, and AD are in line with previous studies (Perneczky et al., 2009; Groot et al., 2018; Ossenkoppele et al., 2020). Although positive effects of education on global cognition and executive functioning were present across all clinical diagnoses, these effects were less pronounced with increasing diagnosis severity. With regard to episodic memory, positive associations with education were merely present in SCD. These results corroborate previous findings among AD-biomarker positive memory clinic patients, where education level was more strongly associated with measures of attention and executive functioning in SCD and MCI when compared to AD, while no associations were found between education and episodic memory (Groot et al., 2018). It has been suggested that clinical deterioration may become too severe at some point in time, accompanied by a subsequent decline of protective mechanisms including the positive effects of education (Stern, 2012; Mungas et al., 2018; van Loenhoud et al., 2019; Soldan et al., 2020). This may also explain why education was not related to episodic memory in MCI and AD patients, as episodic memory loss is considered to be the most pronounced clinical hallmark of these diagnoses and the underlying pathological processes (Albert et al., 2011; McKhann et al., 2011; Veitch et al., 2019).

Although pathological processes overall exacerbate with clinical severity, neuropathological burden remains highly variable within a particular clinical diagnosis (Boyle et al., 2017; Mehta and Schneider, 2021). Previous studies among individuals with normal cognitive functions, MCI, and AD demonstrated that positive effects of education on cognitive performance declined with increased neuropathological burden, sometimes even provoking worse cognitive outcomes (Perneczky et al., 2009; Mungas et al., 2018, 2021; Zahodne et al., 2019). Our findings add to these prior studies by showing that neuropathological burden may differentially alter the association between education and cognition in SCD and MCI. More specifically, we only observed positive effects of education on cognition in those with relatively lower levels of neuropathology in SCD, while in MCI these associations were found in individuals with relatively higher levels of neuropathology. As our results did not survive corrections for multiple comparisons and did not remain significant in several sensitivity analyses, however, cautious interpretation is warranted. Interestingly, similar changes in the direction of effects were observed in a recent study that compared the effects of a composite measure of education and verbal intelligence on episodic memory at different degrees of gray matter atrophy between young-old and old-old participants in a normal elderly cohort (Kwak et al., 2020). Differences in neuropathological burden across seemingly similar populations thus may influence the observed education-cognition associations, conversely contributing to discrepancies in findings across studies (Ewers, 2020; Soldan et al., 2020). More specifically, it has been suggested that the positive effects of education initially emerge as a function of neuropathology, plateau, and subsequently decline (Gregory et al., 2017; Staekenborg et al., 2020). For example, contrarily to our study results and prior findings (Groot et al., 2018), another recent study demonstrated that education more strongly related to cognitive performance in AD relative to SCD and MCI, although these effects diminished in the most severely affected AD patients (Staekenborg et al., 2020).

Several mechanisms have been proposed to explain the benefits of education in both healthy and pathological aging (Fratiglioni et al., 2020). It has been suggested that education contributes to an increased resistance for neurodegenerative processes (i.e., brain maintenance; Noble et al., 2012; Chen et al., 2019; Steffener, 2021). However, this does not explain why individuals with higher levels of education show better cognitive performance at similar levels of neuropathology or demonstrate relatively more neuropathological burden in clinical samples (Stern, 2012; Soldan et al., 2020). Furthermore, a recent large-scale longitudinal study found that education related to an initial advantage in structural properties of the brain instead, and not to different rates in neural decline (Nyberg et al., 2021). Education may thus contribute to a stable advantage, where relatively more neurodegeneration is required before the threshold is reached where cognitive dysfunctions start to emerge (i.e., brain reserve; Cabeza et al., 2018; Stern et al., 2020). In line with this, previous studies associated education with better cognitive functions in healthy aging (Lövdén et al., 2020; Seblova et al., 2020) as well as in SCD, MCI, and AD (Groot et al., 2018; Staekenborg et al., 2020). Moreover, it has been hypothesized that education facilitates cognitive functions by promoting cognitive reserve (CR; Stern, 2012; Stern et al., 2020). CR refers to the ability to compensate for the deteriorative effects of neuropathological processes through the recruitment of existing neural networks and/or compensatory processes *via* alternative networks (Stern, 2012; Cabeza et al., 2018; Soldan et al., 2020; Stern et al., 2020). However, the contribution of education to CR is under debate, as previous longitudinal and cross-sectional studies have not consistently demonstrated that higher levels of education concur with relatively decreased rates of age- or pathology-related cognitive decline over time (Wilson et al., 2019; Ewers, 2020; Lövdén et al., 2020; Seblova et al., 2020). Lastly, it has to be noted that higher levels of cognitive functioning themselves could facilitate the likelihood of an individual completing higher levels of education (Peng and Kievit, 2020). Therefore, the precise underlying mechanisms and direction of effects between education and cognitive performance remain to be elucidated.

The present study has several strengths and limitations. While earlier studies mostly focused on a single marker of neuropathology (Perneczky et al., 2009; Teipel et al., 2009; Mortamais et al., 2014; Soldan et al., 2015; Kwak et al., 2020), we combined multiple visual MRI ratings into a single measure reflective of brain-wide pathology. Nevertheless, these approaches are restricted in terms of the spatial characterization of neurodegenerative processes (Jagust, 2018). Voxel-wise analyses (Ledig et al., 2018), connectivity-based approaches (Berron et al., 2020), or data-driven techniques could help to further delineate neuropathological patterns that may better explain individual variations in cognitive performance (Verdi et al., 2021). Furthermore, although we examined a representative sample of patients referred to a memory clinic in the Netherlands, increasing the external validity of our results, causal inference is impossible due to the lack of longitudinal data, and our cross-sectional design did not allow the investigation of the effects of education on disease progression across the different syndromes. Lastly, we emphasize that our findings require replication in larger cohort studies, given that the neuropathology-dependent effects of education did not survive corrections for multiple comparisons. An increased understanding of such dynamics is not only critical to understand healthy cognitive aging (Cabeza et al., 2018; Stern et al., 2020), but may also aid in the development of individualized prevention or intervention strategies and prognostic models of cognitive decline (Fratiglioni et al., 2020; Livingston et al., 2020; Soldan et al., 2020; Anatürk et al., 2021). Future research should ideally incorporate longitudinal, multi-modal MRI measures to determine how education-cognition associations vary as a function of neuropathology in SCD, MCI, and AD.

In conclusion, we further characterized the extent to which education continues to benefit cognitive performance depending on different disease stages: across and within SCD, MCI, and AD. Generally, the positive effects of education were most strongly pronounced in individuals with SCD and diminished with diagnosis severity. Within a particular diagnosis, however, an increased degree of neuropathological burden does not necessarily imply a reduction of effects. Altogether, our findings highlight the complex dynamics between education and its protective effects on cognitive functions, and the importance of taking into account the diverse dimensions of an individual’s disease severity to understand such associations.

## Data Availability Statement

The data analyzed in this study is subject to the following licenses/restrictions: The datasets analyzed for this study are available upon reasonable request. Requests to access these datasets should be directed to JO, joukje.oosterman@donders.ru.nl.

## Ethics Statement

Ethical review and approval was not required for the study on human participants in accordance with the local legislation and institutional requirements. Written informed consent was not provided because all data were collected as part of routine clinical assessments between 2004 and 2015 and were stored in an in-house clinical database in a pseudononymous manner, compliant with the then-current EU Data Protection Directive 95/46/EC. Data extraction from that database for this study was done by EJO, in compliance with the EU General Data Protection Regulation 2016/679, resulting in a fully anonymous database for further analysis.

## Author Contributions

MJ drafted the main manuscript and prepared all figures and tables. JO, EO, JC, and RK conceived the study and acquired all data. MJ, JO, and LG designed the present study and analyzed all the data. All authors revised the manuscript for intellectual content.

## Conflict of Interest

The authors declare that the research was conducted in the absence of any commercial or financial relationships that could be construed as a potential conflict of interest.

## Publisher’s Note

All claims expressed in this article are solely those of the authors and do not necessarily represent those of their affiliated organizations, or those of the publisher, the editors and the reviewers. Any product that may be evaluated in this article, or claim that may be made by its manufacturer, is not guaranteed or endorsed by the publisher.

## Footnotes

1
https://www.R-project.org

## References

[B1] Albert M. S. DeKosky S. T. Dickson D. Dubois B. Feldman H. H. Fox N. C., et al. (2011). The diagnosis of mild cognitive impairment due to Alzheimer’s disease: recommendations from the National Institute on Aging-Alzheimer’s Association workgroups on diagnostic guidelines for Alzheimer’s disease. *Alzheimers Dement.* 7 270–279. 10.1016/j.jalz.2011.03.008 21514249 PMC3312027

[B2] Allen M. Poggiali D. Whitaker K. Marshall T. van Langen J. Kievit R. (2021). Raincloud plots: a multi-platform tool for robust data visualization [version 2; peer review: 2 approved]. *Wellcome Open Res.* 4:63. 10.12688/wellcomeopenres.15191.2 PMC6480976 31069261

[B3] Anatürk M. Patel R. Georgiopoulos G. Newby D. Topiwala A. de Lange A.-M. G. (2021). Development and validation of a novel dementia risk score in the UK biobank cohort. [Preprint]. *PsyArXiv* 10.31234/osf.io/5yvjr PMC10577770 37603383

[B4] APA. (2000). *Diagnostic and Statistical Manual of Mental Disorders, 4th edition, Text Revision (DSM-IV-TR).* Washington, DC: American Psychiatric Association.

[B5] Bauer D. J. Curran P. J. (2005). Probing interactions in fixed and multilevel regression: inferential and graphical techniques. *Multivariate Behav. Res.* 40 373–400. 10.1207/s15327906mbr4003_5 26794689

[B6] Berron D. van Westen D. Ossenkoppele R. Strandberg O. Hansson O. (2020). Medial temporal lobe connectivity and its associations with cognition in early Alzheimer’s disease. *Brain* 143 1233–1248. 10.1093/brain/awaa068 32252068 PMC7174043

[B7] Boyle P. A. Yang J. Yu L. Leurgans S. E. Capuano A. W. Schneider J. A. (2017). Varied effects of age-related neuropathologies on the trajectory of late life cognitive decline. *Brain* 140 804–812. 10.1093/brain/aww341 28082297 PMC5837473

[B8] Cabeza R. Albert M. Belleville S. Craik F. I. Duarte A. Grady C. L. (2018). Maintenance, reserve and compensation: the cognitive neuroscience of healthy ageing. *Nat. Rev. Neurosci.* 19 701–710. 10.1038/s41583-018-0068-2 30305711 PMC6472256

[B9] Chen Y. Lv C. Li X. Zhang J. Chen K. Liu Z. (2019). The positive impacts of early-life education on cognition, leisure activity, and brain structure in healthy aging. *Aging* 11 4923–4942. 10.18632/aging.102088 31315089 PMC6682517

[B10] Coen R. F. McCarroll K. Casey M. McNulty H. Laird E. Molloy A. M. (2016). The Frontal Assessment Battery: normative Performance in a Large Sample of Older Community-Dwelling Hospital Outpatient or General Practitioner Attenders. *J. Geriatr. Psychiatry Neurol.* 29 338–343. 10.1177/0891988716666381 27647791

[B11] Cohen J. (2013). *Statistical Power Analysis For The Behavioral Sciences.* Cambridge, Massachusetts: Academic press.

[B12] de Jonghe J. F. Krijgsveld S. Staverman K. Lindeboom J. Kat M. G. (1994). Differentiation between dementia and functional psychiatric disorders in a geriatric ward of a general psychiatric hospital using the ‘Amsterdam Dementia-Screening Test’. *Ned. Tijdschr. Geneesk.* 138, 1668–1673. 8090234

[B13] de Vent N. R. Agelink van Rentergem J. A. Schmand B. A. Murre J. M. J. Andi Consortium Huizenga H. M. (2016). Advanced Neuropsychological Diagnostics Infrastructure (ANDI): a Normative Database Created from Control Datasets. *Front. Psychol.* 7:1601. 10.3389/fpsyg.2016.01601 27812340 PMC5071354

[B14] Dubois B. Slachevsky A. Litvan I. Pillon B. (2000). The FAB: a frontal assessment battery at bedside. *Neurology* 55, 1621–1626. 10.1212/wnl.55.11.1621 11113214

[B15] Ewers M. (2020). Reserve in Alzheimer’s disease: update on the concept, functional mechanisms and sex differences. *Curr. Opin. Psychiatry* 33 178–184. 10.1097/YCO.0000000000000574 31789678

[B16] Fazekas F. Chawluk J. B. Alavi A. Hurtig H. I. Zimmerman R. A. (1987). MR signal abnormalities at 1.5 T in Alzheimer’s dementia and normal aging. *Am. J. Roentgenol* 149 351–356. 10.2214/ajr.149.2.351 3496763

[B17] Folstein M. F. Folstein S. E. McHugh P. R. (1975). “Mini-mental state”: a practical method for grading the cognitive state of patients for the clinician. *J. Psychiatr Res.* 12, 189–198. 10.1093/schbul/13.2.261 1202204

[B18] Fratiglioni L. Marseglia A. Dekhtyar S. (2020). Ageing without dementia: can stimulating psychosocial and lifestyle experiences make a difference? *Lancet Neurol.* 19 533–543. 10.1016/S1474-4422(20)30039-9 32470425

[B19] Gouw A. Van der Flier W. Van Straaten E. Barkhof F. Ferro J. Baezner H. (2006). Simple versus complex assessment of white matter hyperintensities in relation to physical performance and cognition: the LADIS study. *J. Neurol.* 253 1189–1196. 10.1007/s00415-006-0193-5 16998647

[B20] Gregory S. Long J. D. Klöppel S. Razi A. Scheller E. Minkova L. (2017). Operationalizing compensation over time in neurodegenerative disease. *Brain* 140 1158–1165. 10.1093/brain/awx022 28334888 PMC5382953

[B21] Groot C. van Loenhoud A. C. Barkhof F. van Berckel B. N. Koene T. Teunissen C. C. (2018). Differential effects of cognitive reserve and brain reserve on cognition in Alzheimer disease. *Neurology* 90 e149–e156. 10.1212/WNL.0000000000004802 29237798

[B22] Jack C. R. Bennett D. A. Blennow K. Carrillo M. C. Dunn B. Haeberlein S. B. (2018). NIA-AA research framework: toward a biological definition of Alzheimer’s disease. *Alzheimers Dement.* 14 535–562. 10.1016/j.jalz.2018.02.018 29653606 PMC5958625

[B23] Jagust W. (2018). Imaging the evolution and pathophysiology of Alzheimer disease. *Nat. Rev. Neurosci.* 19 687–700. 10.1038/s41583-018-0067-3 30266970 PMC7032048

[B24] Kwak S. Shin M. Kim H. Cho B. Ha J. H. Han G. (2020). Moderating effect of cognitive reserve on the association between grey matter atrophy and memory varies with age in older adults. *Psychogeriatrics* 20 87–95. 10.1111/psyg.12460 31069884 PMC7003838

[B25] Ledig C. Schuh A. Guerrero R. Heckemann R. A. Rueckert D. (2018). Structural brain imaging in Alzheimer’s disease and mild cognitive impairment: biomarker analysis and shared morphometry database. *Sci. Rep.* 8:11258. 10.1038/s41598-018-29295-9 30050078 PMC6062561

[B26] Lindeboom J. Schmand B. Tulner L. Walstra G. Jonker C. (2002). Visual association test to detect early dementia of the Alzheimer type. *J. Neurol. Neursurg. Psychiatry* 73, 126–133. 10.1136/jnnp.73.2.126 12122168 PMC1737993

[B27] Livingston G. Huntley J. Sommerlad A. Ames D. Ballard C. Banerjee S. (2020). Dementia prevention, intervention, and care: 2020 report of the Lancet Commission. *Lancet* 396 413–446. 10.1016/S0140-6736(20)30367-6 32738937 PMC7392084

[B28] Lövdén M. Fratiglioni L. Glymour M. M. Lindenberger U. Tucker-Drob E. M. (2020). Education and cognitive functioning across the life span. *Psychol. Sci. Public Interest* 21 6–41. 10.1177/1529100620920576 32772803 PMC7425377

[B29] Lüdecke D. (2021). *sjPlot**: Data Visualization for Statistics in Social Science. R package version 2.8.7* [Online]. Available Online at: https://CRAN.R-project.org/package=sjPlot (accessed March 11, 2021).

[B30] McKhann G. M. Knopman D. S. Chertkow H. Hyman B. T. Jack C. R. Jr. Kawas C. H. (2011). The diagnosis of dementia due to Alzheimer’s disease: recommendations from the National Institute on Aging-Alzheimer’s Association workgroups on diagnostic guidelines for Alzheimer’s disease. *Alzheimers Dement.* 7 263–269. 10.1016/j.jalz.2011.03.005 21514250 PMC3312024

[B31] Mehta R. I. Schneider J. A. (2021). What is ‘Alzheimer’s disease’? The neuropathological heterogeneity of clinically defined Alzheimer’s dementia. *Curr. Opin. Neurol.* 34 237–245. 10.1097/WCO.0000000000000912 33591030

[B32] Mortamais M. Portet F. Brickman A. M. Provenzano F. A. Muraskin J. Akbaraly T. N. (2014). Education modulates the impact of white matter lesions on the risk of mild cognitive impairment and dementia. *Am. J. Geriatr. Psychiatry* 22 1336–1345. 10.1016/j.jagp.2013.06.002 24021219 PMC4143478

[B33] Mungas D. Fletcher E. Gavett B. E. Widaman K. Zahodne L. B. Hohman T. J. (2021). Comparison of Education and Episodic Memory as Modifiers of Brain Atrophy Effects on Cognitive Decline: implications for Measuring Cognitive Reserve. *J. Int. Neuropsychol Soc.* 27 401–411. 10.1017/S1355617720001095 33455611 PMC8137673

[B34] Mungas D. Gavett B. Fletcher E. Farias S. T. DeCarli C. Reed B. (2018). Education amplifies brain atrophy effect on cognitive decline: implications for cognitive reserve. *Neurobiol. Aging* 68 142–150. 10.1016/j.neurobiolaging.2018.04.002 29798764 PMC5993638

[B35] Noble K. G. Grieve S. M. Korgaonkar M. S. Engelhardt L. E. Griffith E. Y. Williams L. M. (2012). Hippocampal volume varies with educational attainment across the life-span. *Front. Hum. Neurosci.* 6:307. 10.3389/fnhum.2012.00307 23162453 PMC3494123

[B36] Nyberg L. Magnussen F. Lundquist A. Baaré W. Bartrés-Faz D. Bertram L. (2021). Educational attainment does not influence brain aging. *Proc. Nat. Acad. Sci.U. S. A.* 118:e2101644118. 10.1073/pnas.2101644118 33903255 PMC8106299

[B37] Ossenkoppele R. Lyoo C. H. Jester-Broms J. Sudre C. H. Cho H. Ryu Y. H. (2020). Assessment of Demographic, Genetic, and Imaging Variables Associated With Brain Resilience and Cognitive Resilience to Pathological Tau in Patients With Alzheimer Disease. *JAMA Neurol.* 77 632–642. 10.1001/jamaneurol.2019.5154 32091549 PMC7042808

[B38] Overdorp E. J. Kessels R. P. Claassen J. A. Oosterman J. M. (2014). Cognitive impairments associated with medial temporal atrophy and white matter hyperintensities: an MRI study in memory clinic patients. *Front. Aging Neurosci.* 6:98. 10.3389/fnagi.2014.00098 24904411 PMC4034495

[B39] Pena E. A. Slate E. H. (2006). Global validation of linear model assumptions. *J. Am. Stat. Assoc.* 101 341–354. 10.1198/016214505000000637 20157621 PMC2820257

[B40] Peng P. Kievit R. A. (2020). The development of academic achievement and cognitive abilities: a bidirectional perspective. *Child Dev. Perspect.* 14 15–20. 10.1111/cdep.12352 PMC7613190 35909387

[B41] Perneczky R. Wagenpfeil S. Lunetta K. L. Cupples L. A. Green R. C. DeCarli C. (2009). Education attenuates the effect of medial temporal lobe atrophy on cognitive function in Alzheimer’s disease: the MIRAGE study. *J. Alzheimers Dis.* 17 855–862. 10.3233/JAD-2009-1117 19542606 PMC2868929

[B42] Persson K. Barca M. L. Cavallin L. Brækhus A. Knapskog A.-B. Selbæk G. (2018). Comparison of automated volumetry of the hippocampus using NeuroQuant^®^ and visual assessment of the medial temporal lobe in Alzheimer’s disease. *Acta Radiol.* 59 997–1001. 10.1177/0284185117743778 29172642

[B43] Petersen R. C. Doody R. Kurz A. Mohs R. C. Morris J. C. Rabins P. V. (2001). Current Concepts in Mild Cognitive Impairment. *Arch. Neurol.* 58 1985–1992. 10.1001/archneur.58.12.1985 11735772

[B44] Reitan R. M. (1958). Validity of the trail making test as an indicator of organic brain damage. *Percept Mot. Skills* 8, 271–276. 10.2466/pms.1958.8.3.271

[B45] Salthouse T. A. (2019). Trajectories of normal cognitive aging. *Psychol. Aging* 34 17–24. 10.1037/pag0000288 30211596 PMC6367038

[B46] Scheltens P. Leys D. Barkhof F. Huglo D. Weinstein H. Vermersch P. (1992). Atrophy of medial temporal lobes on MRI in “probable” Alzheimer’s disease and normal ageing: diagnostic value and neuropsychological correlates. *J. Neurol. Neurosurg. Psychiatry* 55 967–972. 10.1136/jnnp.55.10.967 1431963 PMC1015202

[B47] Scheltens P. Pasquier F. Weerts J. G. Barkhof F. Leys D. (1997). Qualitative assessment of cerebral atrophy on MRI: inter-and intra-observer reproducibility in dementia and normal aging. *Eur. Neurol.* 37 95–99. 10.1159/000117417 9058064

[B48] Seblova D. Berggren R. Lövdén M. (2020). Education and age-related decline in cognitive performance: systematic review and meta-analysis of longitudinal cohort studies. *Ageing Res. Rev.* 58:101005. 10.1016/j.arr.2019.101005 31881366

[B49] Soldan A. Pettigrew C. Albert M. (2020). Cognitive Reserve from the Perspective of Preclinical Alzheimer Disease: 2020 Update. *Clin. Geriatr. Med.* 36 247–263. 10.1016/j.cger.2019.11.006 32222300 PMC7837205

[B50] Soldan A. Pettigrew C. Lu Y. Wang M. C. Selnes O. Albert M. (2015). Relationship of medial temporal lobe atrophy, APOE genotype, and cognitive reserve in preclinical A lzheimer’s disease. *Hum. Brain Mapp.* 36 2826–2841. 10.1002/hbm.22810 25879865 PMC4478167

[B51] Staekenborg S. S. Kelly N. Schuur J. Koster P. Scherder E. Tielkes C. E. M. (2020). Education as Proxy for Cognitive Reserve in a Large Elderly Memory Clinic: ‘Window of Benefit’. *J. Alzheimers Dis.* 76 671–679. 10.3233/JAD-191332 32538838

[B52] Steffener J. (2021). Education and age-related differences in cortical thickness and volume across the lifespan. *Neurobiol. Aging* 102 102–110. 10.1016/j.neurobiolaging.2020.10.034 33765423 PMC8126642

[B53] Stern Y. (2012). Cognitive reserve in ageing and Alzheimer’s disease. *Lancet Neurol.* 11 1006–1012. 10.1016/S1474-4422(12)70191-6 23079557 PMC3507991

[B54] Stern Y. Arenaza-Urquijo E. M. Bartrés-Faz D. Belleville S. Cantilon M. Chetelat G. (2020). Whitepaper: defining and investigating cognitive reserve, brain reserve, and brain maintenance. *Alzheimers Dement.* 16 1305–1311. 10.1016/j.jalz.2018.07.219 30222945 PMC6417987

[B55] Teipel S. J. Meindl T. Wagner M. Kohl T. Bürger K. Reiser M. F. (2009). White Matter Microstructure in Relation to Education in Aging and Alzheimer’s Disease. *J. Alzheimers Dis.* 17 571–583. 10.3233/JAD-2009-1077 19433891

[B56] Topiwala A. Suri S. Allan C. Valkanova V. Filippini N. Sexton C. E. (2019). Predicting cognitive resilience from midlife lifestyle and multi-modal MRI: a 30-year prospective cohort study. *PLoS One* 14:e0211273. 10.1371/journal.pone.0211273 30779761 PMC6380585

[B57] UNESCO. (2011). *International Standard Classification of Education (ISCED).* Montreal, Canada: UNESCO-UIS.

[B58] van Loenhoud A. C. van der Flier W. M. Wink A. M. Dicks E. Groot C. Twisk J. (2019). Cognitive reserve and clinical progression in Alzheimer disease: a paradoxical relationship. *Neurology* 93 e334–e346. 10.1212/WNL.0000000000007821 31266904 PMC6669930

[B59] Veitch D. P. Weiner M. W. Aisen P. S. Beckett L. A. Cairns N. J. Green R. C. (2019). Understanding disease progression and improving Alzheimer’s disease clinical trials: recent highlights from the Alzheimer’s Disease Neuroimaging Initiative. *Alzheimers Dement.* 15 106–152. 10.1016/j.jalz.2018.08.005 30321505

[B60] Verdi S. Marquand A. F. Schott J. M. Cole J. H. (2021). Beyond the average patient: how neuroimaging models can address heterogeneity in dementia. *Brain* [Epub Online ahead of print]. 10.1093/brain/awab165 33892488 PMC8634113

[B61] Verhage F. (1964). *Intelligentie en leeftijd: Onderzoek bij Nederlanders van twaalf tot zevenenzeventig jaar.* Netherlands: Van Gorcum.

[B62] Wilson R. S. Yu L. Lamar M. Schneider J. A. Boyle P. A. Bennett D. A. (2019). Education and cognitive reserve in old age. *Neurology* 92 e1041–e1050. 10.1212/WNL.0000000000007036 30728309 PMC6442015

[B63] Ye B. S. Seo S. W. Cho H. Kim S. Y. Lee J. S. Kim E. J. (2013). Effects of education on the progression of early- versus late-stage mild cognitive impairment. *Int. Psychogeriatr.* 25 597–606. 10.1017/s1041610212002001 23207181

[B64] Zahodne L. B. Mayeda E. R. Hohman T. J. Fletcher E. Racine A. M. Gavett B. (2019). The role of education in a vascular pathway to episodic memory: brain maintenance or cognitive reserve? *Neurobiol. Aging* 84 109–118. 10.1016/j.neurobiolaging.2019.08.009 31539647 PMC6960324

[B65] Zhou T. L. Kroon A. A. van Sloten T. T. van Boxtel M. P. Verhey F. R. Schram M. T. (2019). Greater Blood Pressure Variability Is Associated With Lower Cognitive Performance: the Maastricht Study. *Hypertension* 73 803–811. 10.1161/hypertensionaha.118.12305 30739535

