# Supplementary material for: Positive Effects of Education on Cognitive Functioning Depend on Clinical Status and Neuropathological Severity
Source: Front Hum Neurosci. 2021 Sep 9;15:723728. doi: 10.3389/fnhum.2021.723728 (PMC8459869; doi:10.3389/fnhum.2021.723728)
Supplement: Supplementary file 1 [file Data_Sheet_1.docx]

*Supplementary material – Positive effects of education on cognitive functioning varies across clinical severity and neuropathological severity*

**Michelle G. Jansen^1^, Linda Geerligs^1^, Jurgen A.H.R. Claassen^1,2^, Eduard J. Overdorp^3^, Inti Brazil^1^, Roy P.C. Kessels^1,4^, Joukje M. Oosterman^1^**

^1^Donders Institute for Brain, Cognition and Behaviour, Radboud University Nijmegen, Nijmegen, the Netherlands

^2^Department of Geriatric Medicine, Radboud University Medical Center-Radboud Alzheimer Center, Nijmegen, the Netherlands

^3^Department of Psychiatry, Gelre Medical Centre, Zutphen, the Netherlands

^4^Department of Medical Psychology, Radboud University Medical Center-Radboud Alzheimer Center, Nijmegen, the Netherlands

*** Correspondence:**Drs. Michelle Jansen
michelle.jansen@donders.ru.nl

**Overview of supplementary material**

[Supplementary Table A. Characteristics for the individual (sub-)test scores. 3](#_Toc78295819)

[Supplementary Table B. Effects of education on cognitive performance in SCD, MCI, and AD 4](#_Toc78295820)

[Supplementary Table C. Sensitivity analysis with norm scores. 6](#_Toc78295821)

[Supplementary Table D. Sensitivity analysis without TMT ratio and delayed recall 8](#_Toc78295822)

[Supplementary Table E. Sensitivity analysis with MTA in the interaction term. 10](#_Toc78295823)

[Supplementary Table F. Sensitivity analysis with GA in the interaction term. 12](#_Toc78295824)

[Supplementary Table G. Sensitivity analysis with WMH in the interaction term. 14](#_Toc78295825)

[Supplementary Figure A. Effects of education on cognitive domains calculated with norm scores 16](#_Toc78295826)

# Supplementary Table A. Characteristics for the individual (sub-)test scores.

|  | SCD (N = 108) | | | MCI (N = 190) | | | AD (N = 245) | | | p-value |
| --- | --- | --- | --- | --- | --- | --- | --- | --- | --- | --- |
| Neuropsychological test score |  |  | N |  |  | N |  |  | N |  |
| MMSE, total score, median (IQR) | 28 (27-29.25) | | 108 | 27 (25-28) | | 190 | 23 (20-25) | | 245 | <.001^a,b,c,d^ |
| VAT, total score, median (IQR) | 12 (11-12) | | 96 | 7 (4-10) | | 182 | 3 (1-7) | | 229 | <.001^a,b,c,d^ |
| 8-word test, immediate recall, mean (SD) | 27.83 (5.44) | | 94 | 22.88 (4.92) | | 189 | 19.51 (6.08) | | 242 | <.001^a,b,c,d^ |
| 8-word test, delayed recall, median (IQR) | 4 (2-6) | | 94 | 1 (0-2) | | 187 | 0 (0-1) | | 242 | <.001^a,b,c,d^ |
| Fluency, total score occupations, mean (SD) | 12.82 (4.72) | | 106 | 10.28 (4.15) | | 189 | 7.24 (3.85) | | 244 | <.001^a,b,c,d^ |
| Fluency, total score animals, mean (SD) | 17.16 (4.90) | | 106 | 14.09 (4.68) | | 189 | 11.31 (4.51) | | 243 | <.001^a,b,c,d^ |
| FAB, total score, median (IQR) | 16 (14-17) | | 99 | 14 (12-16) | | 160 | 11 9-13 | | 207 | <.001^a,b,c,d^ |
| TMT A, seconds, median (IQR) | 50 (38.50-59) | | 91 | 58 (48-79.50) | | 135 | 70 (56.50-101) | | 111 | <.001^a,b,c,d^ |
| TMT ratio (B/A), seconds, median (IQR) | 2.39 (2.02-3.10) | | 91 | 3.10 (2.24-4.01) | | 135 | 3.78 (2.66-5.52) | | 111 | <.001^a,b,c,d^ |

Abbreviations: SCD = subjective cognitive decline; MCI = mild cognitive impairment; AD = Alzheimer’s disease dementia; MMSE = Mini Mental State Exam; VAT = Visual Association Test; FAB = Frontal Assessment Battery; TMT = Trail Making Test; IQR = interquartile range; SD = standard deviation. P-values displayed are uncorrected. Univariate tests were used to investigate whether the scores differed per diagnostic group, using analysis of variance (ANOVA), Mann-Whitney U test, and Kruskall-Wallis test, where appropriate.
^a^Group contrast, surviving FDR-correction for multiple comparisons

^b^Significant SCD vs. MCI comparison after FDR-corrections

^c^Significant SCD vs. AD comparison after FDR-corrections

^d^Significant MCI vs. AD comparison after FDR-corrections

# Supplementary Table B. Effects of education on cognitive performance in SCD, MCI, and AD

|  | SCD |  | MCI |  | AD |  |
| --- | --- | --- | --- | --- | --- | --- |
|  | Model 1 | Model 2 | Model 1 | Model 2 | Model 1 | Model 2 |
| Global cognition |  |  |  |  |  |  |
|  |  |  |  |  |  |  |
| Constant | 0.00 (-0.15, 0.15) | 0.01 (-0.14, 0.16) | 0.00 (-0.13, 0.13) | -0.001 (-0.13, 0.13) | 0.00 (-0.12, 0.12) | 0.001 (-0.12, 0.12) |
| Age | -0.06 (-0.25, 0.13) | -0.05 (-0.25, 0.14) | -0.24^***^ (-0.38, -0.10) | -0.24^***^ (-0.38, -0.10) | -0.07 (-0.20, 0.05) | -0.07 (-0.20, 0.06) |
| Sex | -0.05 (-0.20, 0.11) | -0.04 (-0.20, 0.11) | 0.06 (-0.07, 0.20) | 0.07 (-0.07, 0.20) | -0.07 (-0.20, 0.05) | -0.07 (-0.20, 0.05) |
| Neuropathology | -0.15 (-0.34, 0.04) | -0.15 (-0.34, 0.04) | 0.12^*^ (-0.02, 0.26) | 0.12^*^ (-0.02, 0.27) | -0.13^**^ (-0.26, -0.005) | -0.14^**^ (-0.26, -0.01) |
| Education | 0.59^***^ (0.44, 0.75) | 0.59^***^ (0.44, 0.75) | 0.38^***^ (0.24, 0.51) | 0.38^***^ (0.25, 0.51) | 0.21^***^ (0.09, 0.34) | 0.22^***^ (0.09, 0.34) |
| Education* Neuropathology |  | -0.11 (-0.27, 0.06) |  | 0.03 (-0.11, 0.17) |  | -0.03 (-0.16, 0.09) |
| Observations | 108 | 108 | 190 | 190 | 245 | 245 |
| R^2^ | 0.38 | 0.39 | 0.18 | 0.18 | 0.09 | 0.09 |
| Adjusted R^2^ | 0.36 | 0.36 | 0.17 | 0.16 | 0.07 | 0.07 |
| Episodic memory |  |  |  |  |  |  |
| Constant | 0.005 (-0.17, 0.18) | 0.02 (-0.15, 0.20) | 0.00 (-0.14, 0.14) | -0.01 (-0.14, 0.13) | -0.00 (-0.13, 0.13) | 0.004 (-0.12, 0.13) |
| Age | -0.12 (-0.35, 0.11) | -0.11 (-0.33, 0.12) | -0.20^***^ (-0.34, -0.05) | -0.20^***^ (-0.34, -0.05) | -0.05 (-0.18, 0.09) | -0.04 (-0.18, 0.09) |
| Sex | -0.04 (-0.23, 0.14) | -0.02 (-0.20, 0.16) | -0.14^*^ (-0.28, -0.001) | -0.13^*^ (-0.27, 0.01) | -0.05 (-0.18, 0.08) | -0.05 (-0.17, 0.08) |
| Neuropathology | -0.04 (-0.26, 0.18) | -0.05 (-0.26, 0.17) | 0.15^*^ (0.0003, 0.29) | 0.17^**^ (0.02, 0.32) | -0.03 (-0.17, 0.10) | -0.04 (-0.17, 0.10) |
| Education | 0.37^***^ (0.19, 0.55) | 0.37^***^ (0.19, 0.54) | 0.16^**^ (0.02, 0.30) | 0.17^**^ (0.04, 0.31) | 0.06 (-0.07, 0.19) | 0.07 (-0.06, 0.20) |
| Education* Neuropathology |  | -0.23^**^ (-0.42, -0.04) |  | 0.15^**^ (0.01, 0.30) |  | -0.08 (-0.21, 0.05) |
| Observations | 106 | 106 | 190 | 190 | 245 | 245 |
| R^2^ | 0.15 | 0.20 | 0.10 | 0.12 | 0.01 | 0.02 |
| Adjusted R^2^ | 0.12 | 0.16 | 0.08 | 0.10 | -0.005 | -0.003 |
| Executive functions |  |  |  |  |  |  |
| Constant | 0.00 (-0.15, 0.15) | 0.002 (-0.15, 0.15) | 0.00 (-0.13, 0.13) | -0.0000 (-0.13, 0.13) | 0.002 (-0.12, 0.12) | 0.002 (-0.12, 0.12) |
| Age | -0.05 (-0.24, 0.14) | -0.05 (-0.24, 0.15) | -0.18^**^ (-0.32, -0.03) | -0.18^**^ (-0.32, -0.03) | -0.11^*^ (-0.24, 0.01) | -0.12^*^ (-0.24, 0.01) |
| Sex | -0.01 (-0.16, 0.14) | -0.01 (-0.16, 0.15) | 0.19^***^ (0.06, 0.33) | 0.19^***^ (0.06, 0.33) | -0.02 (-0.14, 0.10) | -0.02 (-0.14, 0.10) |
| Neuropathology | -0.14 (-0.34, 0.05) | -0.14 (-0.34, 0.05) | 0.03 (-0.12, 0.17) | 0.03 (-0.12, 0.17) | -0.09 (-0.22, 0.04) | -0.09 (-0.22, 0.04) |
| Education | 0.62^***^ (0.46, 0.77) | 0.62^***^ (0.46, 0.77) | 0.34^***^ (0.20, 0.47) | 0.34^***^ (0.20, 0.47) | 0.31^***^ (0.19, 0.43) | 0.31^***^ (0.19, 0.43) |
| Education* Neuropathology |  | -0.03 (-0.19, 0.13) |  | -0.002 (-0.14, 0.14) |  | 0.02 (-0.10, 0.14) |
| Observations | 108 | 108 | 190 | 190 | 244 | 244 |
| R^2^ | 0.40 | 0.40 | 0.15 | 0.15 | 0.13 | 0.13 |
| Adjusted R^2^ | 0.37 | 0.37 | 0.13 | 0.13 | 0.12 | 0.11 |

Abbreviations: SCD = subjective cognitive decline; MCI = Mild Cognitive Impairment; AD = Alzheimer’s disease dementia. Values represent standardized coefficients (β) and corresponding 95% confidence interval (CI). *p<0.1; **p<.05; ***p<0.01.

# Supplementary Table C. Sensitivity analysis with norm scores.

|  | SCD |  | MCI |  | AD |  |
| --- | --- | --- | --- | --- | --- | --- |
|  | Model 1 | Model 2 | Model 1 | Model 2 | Model 1 | Model 2 |
| Global cognition |  |  |  |  |  |  |
|  |  |  |  |  |  |  |
| Constant | 0.00 (-0.17, 0.17) | 0.01 (-0.16, 0.17) | 0.00 (-0.14, 0.14) | -0.001 (-0.14, 0.14) | 0.00 (-0.12, 0.12) | 0.0004 (-0.12, 0.12) |
| Age | 0.11 (-0.10, 0.32) | 0.12 (-0.09, 0.33) | 0.03 (-0.12, 0.19) | 0.03 (-0.12, 0.19) | 0.19^***^ (0.06, 0.32) | 0.19^***^ (0.06, 0.32) |
| Sex | -0.05 (-0.22, 0.12) | -0.04 (-0.21, 0.13) | 0.04 (-0.10, 0.19) | 0.04 (-0.10, 0.19) | -0.05 (-0.18, 0.08) | -0.05 (-0.18, 0.08) |
| Neuropathology | -0.15 (-0.35, 0.06) | -0.15 (-0.36, 0.06) | 0.08 (-0.07, 0.23) | 0.09 (-0.07, 0.24) | -0.08 (-0.22, 0.05) | -0.08 (-0.22, 0.05) |
| Education | 0.48^***^ (0.31, 0.65) | 0.48^***^ (0.31, 0.65) | 0.16^**^ (0.02, 0.30) | 0.16^**^ (0.02, 0.31) | 0.01 (-0.12, 0.14) | 0.01 (-0.12, 0.14) |
| Education* Neuropathology |  | -0.13 (-0.30, 0.05) |  | 0.02 (-0.13, 0.17) |  | -0.01 (-0.14, 0.12) |
| Observations | 108 | 108 | 190 | 190 | 245 | 245 |
| R^2^ | 0.25 | 0.27 | 0.04 | 0.04 | 0.03 | 0.03 |
| Adjusted R^2^ | 0.23 | 0.23 | 0.02 | 0.01 | 0.02 | 0.01 |
| Episodic memory |  |  |  |  |  |  |
| Constant | -0.01 (-0.19, 0.18) | 0.01 (-0.17, 0.19) | 0.00 (-0.14, 0.14) | -0.01 (-0.15, 0.13) | 0.00 (-0.11, 0.11) | 0.01 (-0.11, 0.12) |
| Age | 0.18 (-0.05, 0.41) | 0.19^*^ (-0.03, 0.42) | 0.15^**^ (0.004, 0.30) | 0.15^**^ (0.004, 0.30) | 0.44^***^ (0.32, 0.56) | 0.44^***^ (0.32, 0.56) |
| Sex | -0.06 (-0.25, 0.13) | -0.04 (-0.23, 0.15) | -0.16^**^ (-0.30, -0.02) | -0.15^**^ (-0.30, -0.01) | 0.03 (-0.08, 0.15) | 0.03 (-0.08, 0.15) |
| Neuropathology | -0.0001 (-0.22, 0.22) | -0.01 (-0.22, 0.21) | 0.06 (-0.09, 0.21) | 0.08 (-0.07, 0.23) | -0.03 (-0.15, 0.09) | -0.04 (-0.16, 0.08) |
| Education | 0.22^**^ (0.03, 0.40) | 0.21^**^ (0.03, 0.39) | 0.01 (-0.13, 0.16) | 0.02 (-0.12, 0.16) | -0.02 (-0.14, 0.09) | -0.01 (-0.13, 0.11) |
| Education* Neuropathology |  | -0.25^**^ (-0.44, -0.06) |  | 0.11 (-0.05, 0.26) |  | -0.08 (-0.20, 0.03) |
| Observations | 106 | 106 | 190 | 190 | 245 | 245 |
| R^2^ | 0.09 | 0.15 | 0.05 | 0.06 | 0.19 | 0.20 |
| Adjusted R^2^ | 0.06 | 0.10 | 0.03 | 0.04 | 0.18 | 0.18 |
| Executive functions |  |  |  |  |  |  |
| Constant | 0.00 (-0.16, 0.16) | 0.001 (-0.16, 0.16) | 0.00 (-0.14, 0.14) | -0.0000 (-0.14, 0.14) | 0.0002 (-0.13, 0.13) | 0.0000 (-0.13, 0.13) |
| Age | 0.04 (-0.17, 0.25) | 0.04 (-0.17, 0.25) | -0.06 (-0.21, 0.10) | -0.06 (-0.21, 0.10) | 0.07 (-0.07, 0.20) | 0.07 (-0.07, 0.20) |
| Sex | -0.02 (-0.19, 0.14) | -0.02 (-0.19, 0.14) | 0.20^***^ (0.06, 0.35) | 0.20^***^ (0.06, 0.35) | 0.01 (-0.12, 0.14) | 0.01 (-0.12, 0.14) |
| Neuropathology | -0.17 (-0.38, 0.03) | -0.17 (-0.38, 0.03) | 0.01 (-0.14, 0.16) | 0.01 (-0.14, 0.16) | -0.05 (-0.19, 0.08) | -0.05 (-0.19, 0.08) |
| Education | 0.53^***^ (0.37, 0.70) | 0.53^***^ (0.37, 0.70) | 0.17^**^ (0.03, 0.31) | 0.17^**^ (0.03, 0.31) | 0.07 (-0.06, 0.20) | 0.07 (-0.06, 0.20) |
| Education* Neuropathology |  | -0.02 (-0.19, 0.16) |  | 0.001 (-0.15, 0.15) |  | 0.004 (-0.13, 0.14) |
| Observations | 108 | 108 | 190 | 190 | 244 | 244 |
| R^2^ | 0.30 | 0.31 | 0.06 | 0.06 | 0.01 | 0.01 |
| Adjusted R^2^ | 0.28 | 0.27 | 0.04 | 0.03 | -0.01 | -0.01 |

Abbreviations: SCD = subjective cognitive decline; MCI = Mild Cognitive Impairment; AD = Alzheimer’s disease dementia. Values represent standardized coefficients (β) and corresponding 95% confidence interval (CI). *p<0.1; **p<.05; ***p<0.01.

# Supplementary Table D. Sensitivity analysis without TMT ratio and delayed recall

|  | SCD |  | MCI |  | AD |  |
| --- | --- | --- | --- | --- | --- | --- |
|  | Model 1 | Model 2 | Model 1 | Model 2 | Model 1 | Model 2 |
| Episodic memory |  |  |  |  |  |  |
| Constant | 0.004 (-0.17, 0.18) | 0.02 (-0.15, 0.19) | 0.00 (-0.14, 0.14) | -0.003 (-0.14, 0.13) | 0.00 (-0.13, 0.13) | 0.004 (-0.12, 0.13) |
| Age | -0.09 (-0.31, 0.13) | -0.07 (-0.29, 0.14) | -0.17** (-0.32, -0.02) | -0.17** (-0.32, -0.02) | -0.04 (-0.17, 0.10) | -0.03 (-0.17, 0.10) |
| Sex | 0.04 (-0.14, 0.23) | 0.06 (-0.11, 0.24) | -0.08 (-0.22, 0.06) | -0.08 (-0.22, 0.06) | -0.04 (-0.17, 0.09) | -0.04 (-0.17, 0.09) |
| Neuropathology | -0.04 (-0.26, 0.18) | -0.05 (-0.26, 0.16) | 0.15** (0.01, 0.30) | 0.16** (0.01, 0.31) | -0.07 (-0.21, 0.06) | -0.08 (-0.21, 0.05) |
| Education | 0.42*** (0.24, 0.60) | 0.42*** (0.25, 0.60) | 0.21*** (0.07, 0.35) | 0.22*** (0.08, 0.35) | 0.09 (-0.04, 0.21) | 0.09 (-0.03, 0.22) |
| Education* Neuropathology |  | -0.25*** (-0.43, -0.07) |  | 0.07 (-0.08, 0.22) |  | -0.08 (-0.21, 0.05) |
| Observations | 108 | 108 | 190 | 190 | 244 | 244 |
| R2 | 0.40 | 0.40 | 0.17 | 0.17 | 0.14 | 0.14 |
| Adjusted R2 | 0.36 | 0.36 | 0.14 | 0.14 | 0.11 | 0.11 |
| Executive functions |  |  |  |  |  |  |
| Constant | 0.00 (-0.14, 0.14) | 0.01 (-0.14, 0.15) | 0.00 (-0.13, 0.13) | -0.0000 (-0.13, 0.13) | 0.001 (-0.12, 0.12) | 0.002 (-0.12, 0.12) |
| Age | -0.06 (-0.24, 0.12) | -0.05 (-0.24, 0.13) | -0.21*** (-0.35, -0.07) | -0.21*** (-0.35, -0.07) | -0.11* (-0.24, 0.01) | -0.11* (-0.24, 0.01) |
| Sex | -0.08 (-0.22, 0.07) | -0.07 (-0.22, 0.08) | 0.16** (0.02, 0.29) | 0.16** (0.02, 0.29) | -0.03 (-0.15, 0.09) | -0.03 (-0.15, 0.09) |
| Neuropathology | -0.20** (-0.39, -0.02) | -0.21** (-0.39, -0.02) | -0.01 (-0.15, 0.14) | -0.01 (-0.15, 0.14) | -0.14** (-0.26, -0.01) | -0.14** (-0.26, -0.01) |
| Education | 0.62*** (0.48, 0.77) | 0.62*** (0.48, 0.77) | 0.33*** (0.20, 0.47) | 0.33*** (0.20, 0.47) | 0.24*** (0.12, 0.37) | 0.25*** (0.12, 0.37) |
| Education* Neuropathology |  | -0.09 (-0.24, 0.06) |  | -0.02 (-0.16, 0.13) |  | -0.01 (-0.14, 0.11) |
| Observations | 108 | 108 | 190 | 190 | 244 | 244 |
| R2 | 0.45 | 0.46 | 0.15 | 0.15 | 0.11 | 0.11 |
| Adjusted R2 | 0.43 | 0.43 | 0.14 | 0.13 | 0.09 | 0.09 |

Abbreviations: SCD = subjective cognitive decline; MCI = Mild Cognitive Impairment; AD = Alzheimer’s disease dementia. Values represent standardized coefficients (β) and corresponding 95% confidence interval (CI). *p<0.1; **p<.05; ***p<0.01.

# Supplementary Table E. Sensitivity analysis with MTA in the interaction term.

|  | SCD |  | MCI |  | AD |  |
| --- | --- | --- | --- | --- | --- | --- |
|  | Model 1 | Model 2 | Model 1 | Model 2 | Model 1 | Model 2 |
| Global cognition |  |  |  |  |  |  |
|  |  |  |  |  |  |  |
| Constant | 0.00 (-0.15, 0.15) | 0.005 (-0.15, 0.16) | 0.00 (-0.13, 0.13) | -0.01 (-0.14, 0.13) | 0.00 (-0.12, 0.12) | 0.001 (-0.12, 0.12) |
| Age | -0.06 (-0.26, 0.14) | -0.05 (-0.25, 0.15) | -0.24^***^ (-0.38, -0.09) | -0.24^***^ (-0.38, -0.09) | -0.06 (-0.19, 0.07) | -0.06 (-0.19, 0.07) |
| Sex | -0.05 (-0.21, 0.12) | -0.04 (-0.20, 0.12) | 0.07 (-0.07, 0.20) | 0.07 (-0.06, 0.21) | -0.06 (-0.19, 0.07) | -0.06 (-0.19, 0.07) |
| MTA | -0.09 (-0.27, 0.09) | -0.08 (-0.26, 0.10) | 0.09 (-0.05, 0.24) | 0.10 (-0.04, 0.24) | -0.04 (-0.16, 0.09) | -0.04 (-0.17, 0.09) |
| GA | -0.05 (-0.23, 0.13) | -0.07 (-0.25, 0.11) | 0.08 (-0.07, 0.22) | 0.08 (-0.06, 0.23) | -0.08 (-0.21, 0.06) | -0.08 (-0.21, 0.06) |
| WMH | -0.07 (-0.25, 0.12) | -0.05 (-0.24, 0.13) | -0.02 (-0.16, 0.13) | -0.02 (-0.16, 0.12) | -0.10 (-0.23, 0.03) | -0.10 (-0.23, 0.04) |
| Education | 0.59^***^ (0.44, 0.75) | 0.59^***^ (0.43, 0.74) | 0.37^***^ (0.23, 0.50) | 0.37^***^ (0.24, 0.51) | 0.21^***^ (0.08, 0.33) | 0.21^***^ (0.08, 0.34) |
| Education*MTA |  | -0.09 (-0.25, 0.07) |  | 0.06 (-0.08, 0.20) |  | -0.02 (-0.14, 0.11) |
| Observations | 108 | 108 | 190 | 190 | 245 | 245 |
| R^2^ | 0.38 | 0.39 | 0.19 | 0.19 | 0.09 | 0.09 |
| Adjusted R^2^ | 0.34 | 0.35 | 0.16 | 0.16 | 0.07 | 0.06 |
| Episodic memory |  |  |  |  |  |  |
| Constant | 0.01 (-0.17, 0.18) | 0.02 (-0.15, 0.19) | 0.00 (-0.14, 0.14) | -0.01 (-0.15, 0.13) | -0.00 (-0.13, 0.13) | 0.003 (-0.12, 0.13) |
| Age | -0.10 (-0.33, 0.13) | -0.08 (-0.30, 0.15) | -0.21^***^ (-0.36, -0.06) | -0.21^***^ (-0.36, -0.06) | -0.05 (-0.19, 0.08) | -0.05 (-0.19, 0.08) |
| Sex | -0.03 (-0.21, 0.16) | -0.02 (-0.20, 0.17) | -0.14^**^ (-0.28, -0.002) | -0.13^*^ (-0.27, 0.01) | -0.05 (-0.18, 0.08) | -0.05 (-0.18, 0.08) |
| MTA | 0.08 (-0.13, 0.28) | 0.09 (-0.10, 0.29) | 0.07 (-0.08, 0.21) | 0.08 (-0.07, 0.23) | -0.02 (-0.15, 0.12) | -0.02 (-0.15, 0.11) |
| GA | -0.10 (-0.31, 0.10) | -0.16 (-0.36, 0.05) | 0.07 (-0.08, 0.22) | 0.08 (-0.07, 0.23) | -0.07 (-0.21, 0.07) | -0.07 (-0.21, 0.07) |
| WMH | -0.08 (-0.29, 0.13) | -0.05 (-0.25, 0.16) | 0.09 (-0.06, 0.24) | 0.08 (-0.06, 0.23) | 0.06 (-0.08, 0.20) | 0.06 (-0.08, 0.20) |
| Education | 0.37^***^ (0.19, 0.55) | 0.36^***^ (0.18, 0.53) | 0.17^**^ (0.03, 0.31) | 0.18^**^ (0.04, 0.32) | 0.06 (-0.07, 0.19) | 0.07 (-0.06, 0.20) |
| Education*MTA |  | -0.26^***^ (-0.43, -0.08) |  | 0.10 (-0.05, 0.24) |  | -0.04 (-0.17, 0.09) |
| Observations | 106 | 106 | 190 | 190 | 245 | 245 |
| R^2^ | 0.17 | 0.24 | 0.10 | 0.11 | 0.02 | 0.02 |
| Adjusted R^2^ | 0.12 | 0.18 | 0.07 | 0.08 | -0.01 | -0.01 |
| Executive functions |  |  |  |  |  |  |
| Constant | 0.00 (-0.15, 0.15) | -0.001 (-0.15, 0.15) | 0.00 (-0.13, 0.13) | -0.01 (-0.14, 0.13) | 0.002 (-0.12, 0.12) | 0.004 (-0.12, 0.12) |
| Age | -0.05 (-0.25, 0.14) | -0.05 (-0.25, 0.14) | -0.18^**^ (-0.32, -0.03) | -0.18^**^ (-0.32, -0.03) | -0.11^*^ (-0.24, 0.02) | -0.11^*^ (-0.24, 0.02) |
| Sex | -0.01 (-0.17, 0.15) | -0.01 (-0.17, 0.15) | 0.21^***^ (0.07, 0.34) | 0.21^***^ (0.08, 0.35) | -0.01 (-0.13, 0.11) | -0.01 (-0.13, 0.11) |
| MTA | -0.10 (-0.28, 0.07) | -0.11 (-0.28, 0.07) | 0.10 (-0.04, 0.24) | 0.11 (-0.04, 0.25) | 0.002 (-0.12, 0.13) | -0.001 (-0.13, 0.12) |
| GA | -0.01 (-0.18, 0.17) | -0.004 (-0.18, 0.18) | 0.05 (-0.10, 0.19) | 0.06 (-0.09, 0.20) | -0.03 (-0.16, 0.10) | -0.03 (-0.16, 0.10) |
| WMH | -0.07 (-0.25, 0.11) | -0.07 (-0.26, 0.11) | -0.11 (-0.25, 0.04) | -0.11 (-0.25, 0.03) | -0.10 (-0.23, 0.03) | -0.10 (-0.23, 0.03) |
| Education | 0.62^***^ (0.46, 0.77) | 0.62^***^ (0.46, 0.77) | 0.31^***^ (0.18, 0.45) | 0.32^***^ (0.18, 0.45) | 0.31^***^ (0.18, 0.43) | 0.31^***^ (0.18, 0.43) |
| Education*MTA |  | 0.02 (-0.14, 0.17) |  | 0.08 (-0.06, 0.22) |  | -0.02 (-0.14, 0.10) |
| Observations | 108 | 108 | 190 | 190 | 244 | 244 |
| R^2^ | 0.40 | 0.40 | 0.17 | 0.17 | 0.14 | 0.14 |
| Adjusted R^2^ | 0.36 | 0.36 | 0.14 | 0.14 | 0.11 | 0.11 |

Abbreviations: SCD = subjective cognitive decline; MCI = Mild Cognitive Impairment; AD = Alzheimer’s disease dementia. Values represent standardized coefficients (β) and corresponding 95% confidence interval (CI). *p<0.1; **p<.05; ***p<0.01.

# Supplementary Table F. Sensitivity analysis with GA in the interaction term.

|  | SCD |  | MCI |  | AD |  |
| --- | --- | --- | --- | --- | --- | --- |
|  | Model 1 | Model 2 | Model 1 | Model 2 | Model 1 | Model 2 |
| Global cognition |  |  |  |  |  |  |
|  |  |  |  |  |  |  |
| Constant | 0.00 (-0.15, 0.15) | 0.01 (-0.14, 0.16) | 0.00 (-0.13, 0.13) | -0.001 (-0.13, 0.13) | 0.00 (-0.12, 0.12) | 0.0000 (-0.12, 0.12) |
| Age | -0.06 (-0.26, 0.14) | -0.06 (-0.26, 0.13) | -0.24^***^ (-0.38, -0.09) | -0.24^***^ (-0.38, -0.09) | -0.06 (-0.19, 0.07) | -0.06 (-0.19, 0.07) |
| Sex | -0.05 (-0.21, 0.12) | -0.04 (-0.20, 0.13) | 0.07 (-0.07, 0.20) | 0.07 (-0.07, 0.20) | -0.06 (-0.19, 0.07) | -0.06 (-0.19, 0.07) |
| MTA | -0.09 (-0.27, 0.09) | -0.11 (-0.29, 0.07) | 0.09 (-0.05, 0.24) | 0.10 (-0.05, 0.24) | -0.04 (-0.16, 0.09) | -0.04 (-0.16, 0.09) |
| GA | -0.05 (-0.23, 0.13) | -0.01 (-0.20, 0.18) | 0.08 (-0.07, 0.22) | 0.08 (-0.07, 0.22) | -0.08 (-0.21, 0.06) | -0.08 (-0.21, 0.06) |
| WMH | -0.07 (-0.25, 0.12) | -0.09 (-0.27, 0.10) | -0.02 (-0.16, 0.13) | -0.02 (-0.16, 0.13) | -0.10 (-0.23, 0.03) | -0.10 (-0.23, 0.04) |
| Education | 0.59^***^ (0.44, 0.75) | 0.60^***^ (0.44, 0.75) | 0.37^***^ (0.23, 0.50) | 0.37^***^ (0.23, 0.50) | 0.21^***^ (0.08, 0.33) | 0.21^***^ (0.08, 0.33) |
| Education*GA |  | -0.11 (-0.27, 0.04) |  | 0.03 (-0.10, 0.16) |  | -0.002 (-0.13, 0.12) |
| Observations | 108 | 108 | 190 | 190 | 245 | 245 |
| R^2^ | 0.38 | 0.39 | 0.19 | 0.19 | 0.09 | 0.09 |
| Adjusted R^2^ | 0.34 | 0.35 | 0.16 | 0.16 | 0.07 | 0.06 |
| Episodic memory |  |  |  |  |  |  |
| Constant | 0.01 (-0.17, 0.18) | 0.02 (-0.16, 0.20) | 0.00 (-0.14, 0.14) | -0.003 (-0.14, 0.13) | -0.00 (-0.13, 0.13) | 0.001 (-0.12, 0.13) |
| Age | -0.10 (-0.33, 0.13) | -0.10 (-0.34, 0.13) | -0.21^***^ (-0.36, -0.06) | -0.21^***^ (-0.36, -0.06) | -0.05 (-0.19, 0.08) | -0.04 (-0.18, 0.09) |
| Sex | -0.03 (-0.21, 0.16) | -0.01 (-0.20, 0.18) | -0.14^**^ (-0.28, -0.002) | -0.14^**^ (-0.28, -0.002) | -0.05 (-0.18, 0.08) | -0.06 (-0.19, 0.08) |
| MTA | 0.08 (-0.13, 0.28) | 0.05 (-0.16, 0.26) | 0.07 (-0.08, 0.21) | 0.08 (-0.07, 0.23) | -0.02 (-0.15, 0.12) | -0.02 (-0.15, 0.11) |
| GA | -0.10 (-0.31, 0.10) | -0.05 (-0.26, 0.17) | 0.07 (-0.08, 0.22) | 0.08 (-0.07, 0.23) | -0.07 (-0.21, 0.07) | -0.07 (-0.21, 0.07) |
| WMH | -0.08 (-0.29, 0.13) | -0.11 (-0.32, 0.11) | 0.09 (-0.06, 0.24) | 0.09 (-0.06, 0.24) | 0.06 (-0.08, 0.20) | 0.06 (-0.08, 0.20) |
| Education | 0.37^***^ (0.19, 0.55) | 0.38^***^ (0.19, 0.56) | 0.17^**^ (0.03, 0.31) | 0.17^**^ (0.03, 0.31) | 0.06 (-0.07, 0.19) | 0.07 (-0.06, 0.20) |
| Education*GA |  | -0.15 (-0.33, 0.03) |  | 0.13^*^ (-0.003, 0.27) |  | -0.08 (-0.21, 0.05) |
| Observations | 106 | 106 | 190 | 190 | 245 | 245 |
| R^2^ | 0.17 | 0.19 | 0.10 | 0.12 | 0.02 | 0.02 |
| Adjusted R^2^ | 0.12 | 0.13 | 0.07 | 0.09 | -0.01 | -0.01 |
| Executive functions |  |  |  |  |  |  |
| Constant | 0.00 (-0.15, 0.15) | 0.01 (-0.15, 0.16) | 0.00 (-0.13, 0.13) | -0.0002 (-0.13, 0.13) | 0.002 (-0.12, 0.12) | 0.001 (-0.12, 0.12) |
| Age | -0.05 (-0.25, 0.14) | -0.05 (-0.25, 0.14) | -0.18^**^ (-0.32, -0.03) | -0.18^**^ (-0.32, -0.03) | -0.11^*^ (-0.24, 0.02) | -0.12^*^ (-0.25, 0.01) |
| Sex | -0.01 (-0.17, 0.15) | -0.01 (-0.17, 0.15) | 0.21^***^ (0.07, 0.34) | 0.21^***^ (0.07, 0.34) | -0.01 (-0.13, 0.11) | -0.005 (-0.13, 0.12) |
| MTA | -0.10 (-0.28, 0.07) | -0.12 (-0.30, 0.06) | 0.10 (-0.04, 0.24) | 0.10 (-0.04, 0.25) | 0.002 (-0.12, 0.13) | 0.01 (-0.12, 0.13) |
| GA | -0.01 (-0.18, 0.17) | 0.02 (-0.16, 0.20) | 0.05 (-0.10, 0.19) | 0.05 (-0.10, 0.19) | -0.03 (-0.16, 0.10) | -0.03 (-0.16, 0.10) |
| WMH | -0.07 (-0.25, 0.11) | -0.08 (-0.27, 0.10) | -0.11 (-0.25, 0.04) | -0.11 (-0.25, 0.04) | -0.10 (-0.23, 0.03) | -0.11 (-0.24, 0.02) |
| Education | 0.62^***^ (0.46, 0.77) | 0.62^***^ (0.46, 0.77) | 0.31^***^ (0.18, 0.45) | 0.31^***^ (0.18, 0.45) | 0.31^***^ (0.18, 0.43) | 0.30^***^ (0.18, 0.42) |
| Education*GA |  | -0.07 (-0.23, 0.08) |  | 0.01 (-0.12, 0.14) |  | 0.11^*^ (-0.01, 0.24) |
| Observations | 108 | 108 | 190 | 190 | 244 | 244 |
| R^2^ | 0.40 | 0.40 | 0.17 | 0.17 | 0.14 | 0.15 |
| Adjusted R^2^ | 0.36 | 0.36 | 0.14 | 0.13 | 0.11 | 0.12 |

Abbreviations: SCD = subjective cognitive decline; MCI = Mild Cognitive Impairment; AD = Alzheimer’s disease dementia. Values represent standardized coefficients (β) and corresponding 95% confidence interval (CI). *p<0.1; **p<.05; ***p<0.01.

# Supplementary Table G. Sensitivity analysis with WMH in the interaction term.

|  | SCD |  | MCI |  | AD |  |
| --- | --- | --- | --- | --- | --- | --- |
|  | Model 1 | Model 2 | Model 1 | Model 2 | Model 1 | Model 2 |
| Global cognition |  |  |  |  |  |  |
|  |  |  |  |  |  |  |
| Constant | 0.00 (-0.15, 0.15) | -0.0001 (-0.15, 0.15) | 0.00 (-0.13, 0.13) | -0.01 (-0.14, 0.13) | 0.00 (-0.12, 0.12) | -0.002 (-0.12, 0.12) |
| Age | -0.06 (-0.26, 0.14) | -0.06 (-0.26, 0.14) | -0.24^***^ (-0.38, -0.09) | -0.24^***^ (-0.39, -0.10) | -0.06 (-0.19, 0.07) | -0.06 (-0.19, 0.07) |
| Sex | -0.05 (-0.21, 0.12) | -0.05 (-0.21, 0.12) | 0.07 (-0.07, 0.20) | 0.07 (-0.06, 0.20) | -0.06 (-0.19, 0.07) | -0.06 (-0.19, 0.06) |
| MTA | -0.09 (-0.27, 0.09) | -0.09 (-0.27, 0.09) | 0.09 (-0.05, 0.24) | 0.10 (-0.05, 0.24) | -0.04 (-0.16, 0.09) | -0.04 (-0.16, 0.09) |
| GA | -0.05 (-0.23, 0.13) | -0.05 (-0.23, 0.13) | 0.08 (-0.07, 0.22) | 0.08 (-0.07, 0.22) | -0.08 (-0.21, 0.06) | -0.08 (-0.21, 0.06) |
| WMH | -0.07 (-0.25, 0.12) | -0.07 (-0.25, 0.12) | -0.02 (-0.16, 0.13) | -0.02 (-0.16, 0.12) | -0.10 (-0.23, 0.03) | -0.10 (-0.23, 0.03) |
| Education | 0.59^***^ (0.44, 0.75) | 0.59^***^ (0.43, 0.75) | 0.37^***^ (0.23, 0.50) | 0.37^***^ (0.23, 0.50) | 0.21^***^ (0.08, 0.33) | 0.20^***^ (0.08, 0.33) |
| Education*WMH |  | 0.004 (-0.15, 0.16) |  | -0.05 (-0.18, 0.09) |  | -0.04 (-0.17, 0.08) |
| Observations | 108 | 108 | 190 | 190 | 245 | 245 |
| R^2^ | 0.38 | 0.38 | 0.19 | 0.19 | 0.09 | 0.09 |
| Adjusted R^2^ | 0.34 | 0.34 | 0.16 | 0.16 | 0.07 | 0.07 |
| Episodic memory |  |  |  |  |  |  |
| Constant | 0.01 (-0.17, 0.18) | 0.004 (-0.18, 0.18) | 0.00 (-0.14, 0.14) | 0.01 (-0.13, 0.14) | -0.00 (-0.13, 0.13) | -0.01 (-0.13, 0.12) |
| Age | -0.10 (-0.33, 0.13) | -0.11 (-0.34, 0.13) | -0.21^***^ (-0.36, -0.06) | -0.20^**^ (-0.35, -0.05) | -0.05 (-0.19, 0.08) | -0.05 (-0.18, 0.09) |
| Sex | -0.03 (-0.21, 0.16) | -0.03 (-0.22, 0.16) | -0.14^**^ (-0.28, -0.002) | -0.15^**^ (-0.29, -0.005) | -0.05 (-0.18, 0.08) | -0.06 (-0.19, 0.07) |
| MTA | 0.08 (-0.13, 0.28) | 0.07 (-0.14, 0.28) | 0.07 (-0.08, 0.21) | 0.06 (-0.09, 0.21) | -0.02 (-0.15, 0.12) | -0.02 (-0.15, 0.12) |
| GA | -0.10 (-0.31, 0.10) | -0.09 (-0.30, 0.12) | 0.07 (-0.08, 0.22) | 0.07 (-0.08, 0.22) | -0.07 (-0.21, 0.07) | -0.06 (-0.20, 0.07) |
| WMH | -0.08 (-0.29, 0.13) | -0.08 (-0.29, 0.13) | 0.09 (-0.06, 0.24) | 0.09 (-0.06, 0.24) | 0.06 (-0.08, 0.20) | 0.06 (-0.08, 0.20) |
| Education | 0.37^***^ (0.19, 0.55) | 0.37^***^ (0.18, 0.55) | 0.17^**^ (0.03, 0.31) | 0.17^**^ (0.03, 0.31) | 0.06 (-0.07, 0.19) | 0.05 (-0.07, 0.18) |
| Education*WMH |  | 0.06 (-0.12, 0.24) |  | 0.05 (-0.09, 0.19) |  | -0.09 (-0.22, 0.04) |
| Observations | 106 | 106 | 190 | 190 | 245 | 245 |
| R^2^ | 0.17 | 0.17 | 0.10 | 0.11 | 0.02 | 0.02 |
| Adjusted R^2^ | 0.12 | 0.11 | 0.07 | 0.07 | -0.01 | -0.005 |
| Executive functions |  |  |  |  |  |  |
| Constant | 0.00 (-0.15, 0.15) | 0.0004 (-0.15, 0.15) | 0.00 (-0.13, 0.13) | -0.01 (-0.14, 0.13) | 0.002 (-0.12, 0.12) | 0.003 (-0.12, 0.12) |
| Age | -0.05 (-0.25, 0.14) | -0.05 (-0.25, 0.15) | -0.18^**^ (-0.32, -0.03) | -0.18^**^ (-0.33, -0.04) | -0.11^*^ (-0.24, 0.02) | -0.11^*^ (-0.24, 0.02) |
| Sex | -0.01 (-0.17, 0.15) | -0.01 (-0.17, 0.15) | 0.21^***^ (0.07, 0.34) | 0.21^***^ (0.07, 0.34) | -0.01 (-0.13, 0.11) | -0.01 (-0.13, 0.12) |
| MTA | -0.10 (-0.28, 0.07) | -0.10 (-0.28, 0.08) | 0.10 (-0.04, 0.24) | 0.10 (-0.04, 0.25) | 0.002 (-0.12, 0.13) | 0.001 (-0.12, 0.13) |
| GA | -0.01 (-0.18, 0.17) | -0.01 (-0.19, 0.17) | 0.05 (-0.10, 0.19) | 0.05 (-0.10, 0.19) | -0.03 (-0.16, 0.10) | -0.03 (-0.16, 0.10) |
| WMH | -0.07 (-0.25, 0.11) | -0.07 (-0.25, 0.11) | -0.11 (-0.25, 0.04) | -0.11 (-0.25, 0.03) | -0.10 (-0.23, 0.03) | -0.10 (-0.23, 0.03) |
| Education | 0.62^***^ (0.46, 0.77) | 0.62^***^ (0.46, 0.77) | 0.31^***^ (0.18, 0.45) | 0.31^***^ (0.18, 0.45) | 0.31^***^ (0.18, 0.43) | 0.31^***^ (0.18, 0.43) |
| Education*WMH |  | -0.02 (-0.17, 0.13) |  | -0.06 (-0.20, 0.07) |  | 0.01 (-0.11, 0.13) |
| Observations | 108 | 108 | 190 | 190 | 244 | 244 |
| R^2^ | 0.40 | 0.40 | 0.17 | 0.17 | 0.14 | 0.14 |
| Adjusted R^2^ | 0.36 | 0.36 | 0.14 | 0.14 | 0.11 | 0.11 |

Abbreviations: SCD = subjective cognitive decline; MCI = Mild Cognitive Impairment; AD = Alzheimer’s disease dementia. Values represent standardized coefficients (β) and corresponding 95% confidence interval (CI). *p<0.1; **p<.05; ***p<0.01.

# Supplementary Figure A. Effects of education on cognitive domains calculated with norm scores


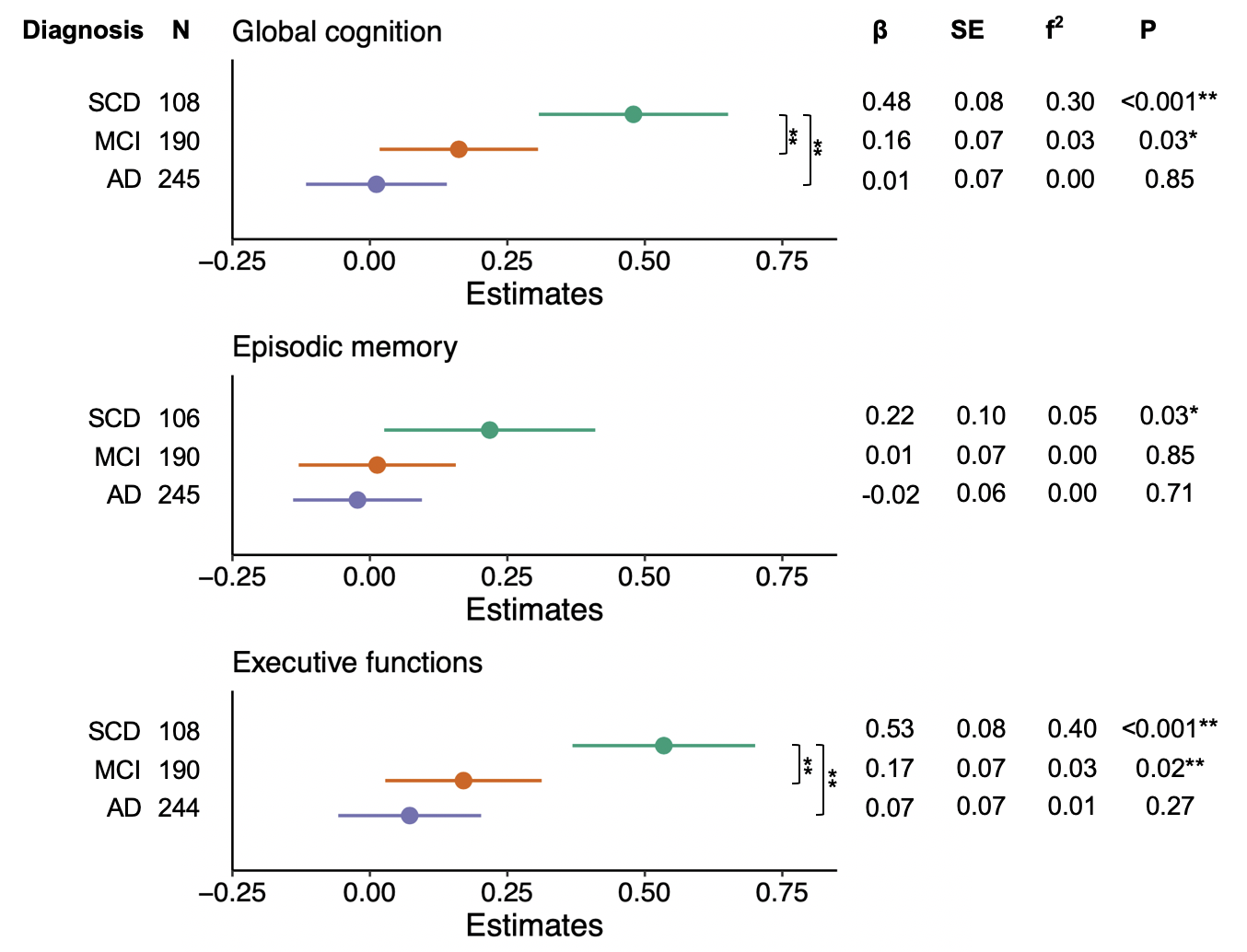


Forest plots indicating the role of education in predicting cognitive performance across diagnosis groups, corrected for age, sex, and neuropathological burden. Effect sizes for the contribution of education were calculated with Cohen’s f2. P-values displayed are uncorrected. Differences in slopes (β) between diagnosis groups were compared using Welch’s t-tests (SCD vs. MCI, SCD vs. AD, MCI vs. AD). Abbreviations: SCD = subjective cognitive decline; MCI = mild cognitive impairment; AD = Alzheimer’s disease dementia.

*Uncorrected p < 0.05

**FDR-corrected p < 0.05
